# Leveraging quantitative systems pharmacology modeling for elranatamab regimen optimization in relapsed or refractory multiple myeloma

**DOI:** 10.1038/s41540-025-00585-z

**Published:** 2025-09-01

**Authors:** Kamrine E. Poels, Mohamed Elmeliegy, Jennifer Hibma, Diane Wang, Cynthia J. Musante, Blerta Shtylla

**Affiliations:** 1 Pharmacometrics & Systems Pharmacology, Pfizer Research & Development, San Diego, CA USA; 2 Clinical Pharmacology, Oncology Research and Development, Pfizer, San Diego, CA USA

**Keywords:** Cancer, Computer modelling, Dynamical systems

## Abstract

Elranatamab, an approved bispecific antibody (BsAb) for relapsed/refractory multiple myeloma, forms an immune synapse between the T-cell CD3 marker and B-cell maturation antigen (BCMA) on myeloma cells. Circulating soluble BCMA (sBCMA) is associated with disease burden and may reduce drug exposure, impacting efficacy. A quantitative systems pharmacology model that captures elranatamab’s mechanism of action and disease dynamics was developed and calibrated to clinical datasets. Simulations explored model uncertainty and inter-patient variability with respect to biological, pharmacologic, and tumor-related components to inform clinical dose-response relationships and evaluate the effect of baseline sBCMA levels on dose and regimen. Model simulations supported 76 mg weekly as the optimal regimen, including in patients with high sBCMA. A left shift in the dose-response curve among virtual responders supported maintenance of efficacy with less frequent dosing. This work exemplifies how mechanistic models may support BsAb dose and regimen justification within the framework of model-informed drug development.

## Introduction

Multiple myeloma (MM) is a genetically heterogenous malignancy of clonal plasma cells that accumulate in the bone marrow and produce abnormal monoclonal immunoglobulin protein (M-protein)^1–3^. Patients with MM often experience relapse and consequently progressively worse outcomes with later lines of therapy, highlighting the need for novel therapeutic agents and targets^1,2,4–6^. B-cell maturation antigen (BCMA) has emerged as a promising target for MM due to its highly selective expression in malignant plasma cells^7^. Several BCMA-targeted therapies, including T-cell–directing/engaging bispecific antibodies (BsAbs), have shown remarkable clinical efficacy in patients with relapsed or refractory MM (RRMM). Elranatamab is a humanized BsAb that engages the CD3 receptor on T cells and BCMA on myeloma cells to induce a selective T-cell response^8,9^. BsAb-mediated T-cell activation results in the secretion of perforin and granzyme B, along with multiple cytokines, such as IFN-γ, IL-2, IL-6, IL-10, and TNF-α, that may further enhance the antitumor response^8–12^. Elranatamab has received accelerated approval for RRMM and is in development for other MM patient populations^13,14^.

BsAbs have a complex exposure-response relationship that depends on many factors, including drug-specific factors (e.g., binding affinity, pharmacokinetic [PK] properties such as absorption, elimination, and distribution), intrinsic activity of the tri-molecular synapse, and system-specific factors (e.g., target expression, effector-target ratio, effector cell concentration, potency)^15,16^. Additionally, MM disease characteristics, such as the presence of soluble BCMA (sBCMA), can reduce the efficacy of therapy by binding to the BsAb and thereby potentially obstructing the formation of a functional trimer complex that triggers cell death and T-cell activation^17,18^. The interplay and downstream impact of these complexities ultimately reduce the ability to identify an efficacious dose range for clinical translation. Poor dose optimization can have a negative impact on patients, including reduced effectiveness, because patients often cannot continue receiving current therapy or receive subsequent therapy due to toxicity. In oncology drug development, maximum tolerated dose–based strategies for clinical dose recommendations have historically been used; however, there is a new focus on a dose-optimization paradigm that identifies doses that maximize response while accounting for the importance of long-term tolerability^19–21^.

Quantitative systems pharmacology (QSP) modeling is a valuable tool to enhance quantitative understanding of the interplay between BsAbs and disease-specific complexities^17,18^. Published models of MM have been developed to answer distinct questions regarding treatment toxicity such as cytokine release syndrome or MM cell interactions with immune cells^19–23^. Betts et al. ^18^ developed a solid tumor model with CD3 BsAb therapy that describes the drug binding to target and T cells and incorporates T-cell distribution to the tumor site. The model, which was developed with preclinical data, has paved the way to provide a quantitative understanding of the complex bell-shaped dose-response curve associated with the CD3-engaging BsAb. Similarly, the work of Hosseini et al. ^22^ illustrated how nonclinical datasets could be used to develop a mechanistically refined model that supported mechanistic rationale for dose priming to mitigate cytokine release syndrome (CRS). This work was later adapted to help characterize the dose response for a phase 1 study of mosunetuzumab in patients with non-Hodgkin lymphoma^23^. These published frameworks of QSP modeling for BsAbs illustrate the impact that these models can deliver when calibrated with nonclinical and early clinical data, thus providing a robust in silico framework that can answer clinical dose/regimen optimization questions through quantitative integration of all data in alignment with project Optimus guidance^24^.

In this work, we outline the development of a QSP model that builds on the work of Betts et al. ^18^ and incorporates tumor dynamics for patients with RRMM and CD3 T- and plasma-cell interactions with elranatamab using data from first-in-human phase 1 (MagnetisMM-1; NCT03269136) and registrational phase 2 MagnetisMM-3 studies (NCT04649359), which evaluated the efficacy and safety of elranatamab monotherapy in patients with RRMM^18,25–27^. Virtual clinical trial simulations of the QSP model were calibrated using multiple efficacy biomarkers (e.g., serum M-protein, serum free light chain [FLC]). Virtual patients and trials were used to explore the dose-response relationship for different patient populations, using baseline (BL) sBCMA levels as a stratification biomarker for the phase 1 MagnetisMM-1 study. Additional data from the phase 2 MagnetisMM-3 study were used to explore further patient response variability across a range of clinically relevant regimens. The QSP model facilitated careful assessment of key MM and immune components and their interplay as various regimens were tested. Simulations demonstrated that, for patients who respond within the first 6 months, efficacy is maintained despite de-escalation of the dosing frequency from weekly to every 2 weeks and then monthly. The QSP model discussed here served as an in-silico hypothesis testing framework to support the regimen for and enhance mechanistic understanding of elranatamab, which may provide advantages to a broad range of patients with RRMM.

## Results

### Overview of model structure and virtual population calibration

**Fig. 1:**
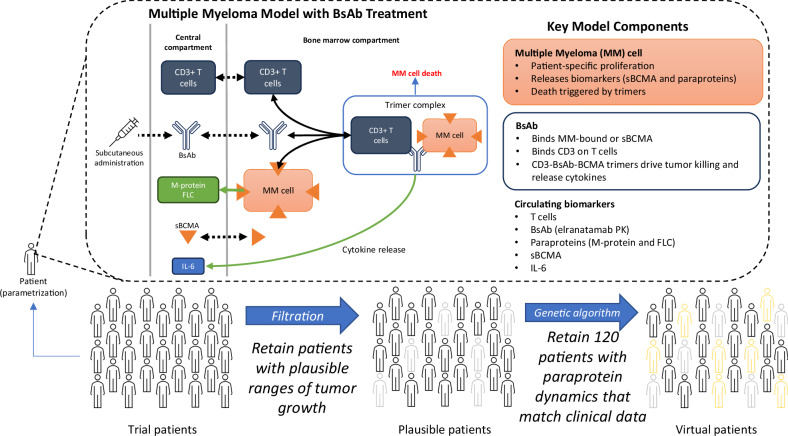
Schema of QSP and virtual population simulation framework. The QSP model describes the dynamic changes in MM cells over time in the BM, which provides a generalized site-of-action compartment. A BsAb engages CD3 receptors on T cells and BCMA receptors on MM cells to form BsAb-CD3-BCMA dimers and trimers. Trimers can facilitate MM cell death, activate T cells, and lead to pro-inflammatory cytokine production that helps attenuate T-cell migration out of the BM compartment. MM cells produce paraproteins such as M-protein and FLC that can be used for assessment of responses in virtual patients. MM cells can shed sBCMA both in the BM and in circulation. A BsAb can bind to sBCMA, as well as T cells in circulation in the central compartment (binding not visualized in the schematic). Model parameters and initial states are varied when the model is initiated, and a trial patient is defined as a non-informed parametrization of the model. From trial patients, we select a population of plausible patients with tumor doubling times that fall within a range supported by the literature. We then select 120 virtual patients from the plausible patient pool such that their summary efficacy endpoints and paraprotein dynamics match those of the elranatamab trial patients. We repeat this step 10 times, selecting different sets of 120 virtual patients. BM bone marrow, BsAb bispecific antibody, FLC free light chain, MM multiple myeloma, PK pharmacokinetics, QSP quantitative systems pharmacology, sBCMA soluble B-cell maturation antigen.

The QSP model consists of several nonlinear ordinary differential equations that link receptor binding with BsAbs to form dimers and trimers with tumor cells and relevant MM biomarkers commonly used to assess clinical response. A schematic of the interactions in the three-compartment model is shown in Fig. 1. The three compartments in the model are: central or circulatory, the bone marrow (BM) or the tumor site, and the peripheral compartment (not shown in Fig. 1). In the bone marrow (BM) compartment, which serves as the primary site of action, a BsAb binds CD3 receptors on T cells and BCMA receptors on MM cells to form BsAb-CD3-BCMA trimers, which in turn initiate tumor cell death^28^. BCMA receptors can be shed and released from the tumor cell’s surface into the BM and central compartment, thereafter referred to as soluble BCMA (sBCMA), in agreement with prior in vivo and clinical studies^10,29,30^. We assume that sBCMA can bind to one arm of the BsAb in the central and BM compartments, forming nonfunctional dimers or trimers, which may ultimately inhibit antitumor efficacy due to a potential drug-sink effect (not shown in the model schema)^18,31–33^. However, when a trimer complex is successfully formed with tumor-bound BCMA, the T cells become activated and trigger cytokine release, pushing the system to a pro-inflammatory state that alters the passage of T cells between compartments via IL-6^34^. Lastly, MM cells shed M-protein and FLC paraproteins into the circulating system^35^. These paraproteins were added to the model since they are used for monitoring therapy response in the clinic^36,37^. Although not shown in the model schema, the BsAb also binds to BCMA, sBCMA, or CD3 to form dimer complexes. More details of the QSP model, including model equations and descriptions of parameters, can be found in the Supplementary Materials.

### QSP virtual population calibration to phase 1 and 2 clinical datasets

We use a virtual population simulation framework with the QSP model to facilitate model calibration to clinical data and assess model prediction uncertainty^38,39^. At a high level, a virtual population (VPop) in this work corresponds to a multistep algorithm for sampling and selecting model parameter sets that yield reasonable model outputs under different treatment regimens. The model was initially simulated with 10,000 random parameter sets and then passed through two filtration/optimization steps that used a genetic algorithm (see Supplementary Fig. 3)^40^ to facilitate: i) selection of plausible patients corresponding to QSP model parametrizations that, upon simulation, produce acceptable ranges of untreated tumor M-protein dynamics and ii) selection of virtual patients through an optimization algorithm that selects subsets of plausible patients probabilistically to match treated tumor-associated paraprotein dynamics obtained from elranatamab studies (see Supplementary Table 4). Thus, each VPop consists of approximately 120 different parametrizations of the QSP model such that their summary statistics (described below) match those observed in MagnetisMM-1 and MagnetisMM-3 subcohorts when simulated under the same schedules administered in the trial. Each of these parametrizations, referred to as a virtual patient, represents a realistic model output.

**Fig. 2:**
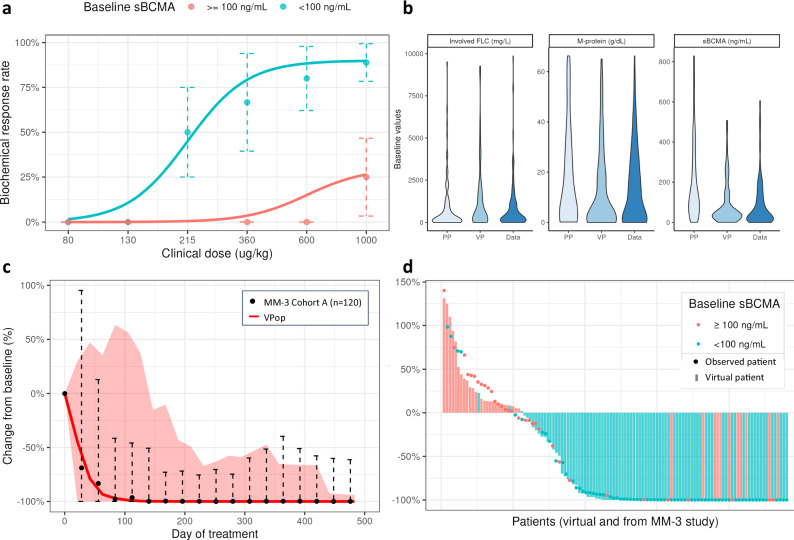
Calibration of model virtual populations to clinical data. **a** Due to its potential role as a drug-sink, sBCMA was identified as a baseline predictor of response. Stratification of patients by baseline sBCMA levels showed a distinction of dose-response curves in the MM-1 study. **b** Efficacy biomarkers and sBCMA are states in the model initialized by sampling patient-specific initial values. The distribution of each variable is fitted to clinical data and used to initiate the plausible patients. After the model is fitted, the density of virtual patients does not show extensive deviation from the distribution of the observed patients (data). **c** Percent change from baseline of paraprotein was used for model calibration across all studies. Median change from baseline and 95% prediction intervals of a VPop are shown in the red solid line and shaded area, respectively, compared to the observed median and 95% coverage of observed values shown in black dots and error bars, respectively. **d** Best biochemical response (i.e., BOR) was also used for model calibration in larger studies. Simulated best response of 120 virtual patients is shown in colored bars, compared with the best biochemical response of 120 patients in MM-3 Cohort A shown in dots colored by baseline sBCMA level. Responses are ranked in ascending order in terms of efficacy. FLC free light chain, MM MagnetisMM, sBCMA soluble B-cell maturation antigen, VPop virtual population.

A subset of nine model parameters was selected to be varied for plausible and virtual populations by using local sensitivity analysis and evidence from the literature about anticipated sources of biological heterogeneity (see Supplementary Table 1 and Supplementary Fig. 1). The parameters identified from the local sensitivity analysis were mostly associated with trimer formation (i.e., receptor densities), tumor killing (i.e., *n*_*kill*_, *α*_*kill*_), and drug resistance (*α*_*resis*_). These nine parameters were sampled from uniform distributions to build 10,000 parametrizations. Next plausible patients were selected from the parametrizations based on evaluation of model-simulated untreated serum M-protein doubling times and cross-checking that they were within clinically sensible published ranges^41,42^ (see Supplementary Fig. 2 for parameter distributions). A schema of the model calibration workflow is shown in Supplementary Fig. 3a. The objective function compared summary statistics of interim VPops to observed summary statistics in clinical data and selected the virtual cohort with the closest fit to clinical data. Specifically, the objective function included three efficacy metrics of serum paraprotein response (see Supplementary Figs. 3b, 4 for details): (1) serum integrated paraprotein dynamics, (2) best overall response (BOR) based on serum biochemical response, and (3) biochemical response rate (BRR) stratified by BL sBCMA levels. The integrated paraprotein was defined as either M-protein or FLC, selected per patient by observation of the serum paraproteins at BL, prioritizing M-protein if it was measurable ( ≥ 0.5 g/dL) and otherwise using FLC as a biomarker of response. A set of 120 virtual patients (corresponding to one VPop) was selected from the plausible patients. More details on the estimation of these metrics are found in the Methods section. The distribution of perturbed parameters from plausible patients to virtual patients remained mostly unchanged, indicating that parameter filtration was not biased (see Supplementary Fig. 2). The BL characteristics of the resulting VPops covered the ranges observed in clinical data, specifically for M-protein, FLC, and sBCMA measurements as shown in Fig. 2b.

Higher levels of sBCMA have been associated with poorer patient outcomes^29,31,32,43^, potentially due to a reduction in target availability for BsAb binding^14^ or being a marker of higher disease burden^44^. Across the doses administered in the MagnetisMM-1 and MagnetisMM-3 studies, objective responses were found to be lower among patients with high sBCMA levels ( ≥ 100 ng/mL) compared with patients with low sBCMA levels ( < 100 ng/mL) at BL (see Fig. 2a). We used 100 ng/mL as the threshold since it was identified as the most significant cutoff that predicted objective response (see Methods for more details). We adjusted for this confounder by calibrating the model to two distinct dose-response curves dependent on BL sBCMA levels.

To facilitate model output calibration to data, we defined a biochemical response as a decrease of ≥50% in serum paraprotein levels that was persistent (i.e., response is maintained for two consecutive observed or simulated tumor assessments). The BRR was estimated from data for some cohorts in MagnetisMM-1 (parts 1 and 2 A) and MagnetisMM-3 (Cohort A) studies, which included different administered doses, stratified by sBCMA levels at BL. Our virtual trial simulations built a cohort of 120 virtual patients that closely matched the estimated BRR of a study cohort when simulated under similar conditions. Simultaneously, within each cohort, median paraprotein dynamics were calibrated to median observed percent change from BL, and the information from each study was weighted by trial size. Fig. 2c illustrates good agreement of median paraprotein dynamics between a fitted VPop and patients from MagnetisMM-3 Cohort A (patients with no prior exposure to BCMA-targeted therapy), and Supplementary Fig. 9 shows that the same VPop appropriately fits a smaller cohort from MM-1. Lastly, BOR from virtual patients was matched to observed BOR, as shown in Fig. 2d, to build a VPop that matched the efficacy summary metrics, such as BRR and median change in paraprotein levels, in the higher-dose cohorts. To fit the VPop’s BOR to the observed BOR, we ranked BORs by decreasing order, split them into 10 groups, and estimated the median of each group. Then, each 10th percentile of a VPop was matched to the corresponding 10th percentile in the data (see Supplementary Fig. 6). We also explored dynamics of other variables that were not used for model calibration but were measurable in the clinic. Among these variables were the concentrations of the therapeutic BsAb and sBCMA. In the clinic, these values are measured using two different assays that report a total concentration of analyte (unbound and bound analyte) or concentration of the analyte in free, or unbound form.

### QSP virtual trials to support understanding of the dose-response relationship for patient subpopulations with different BL sBCMA levels

After calibrating 10 VPops (each with 120 virtual patients), we simulated a dose-escalation virtual trial using doses ranging from 16 mg to 152 mg once weekly (QW). These simulations help strengthen our understanding of the dose-response relationship for the MagnetisMM-1 study as the clinical dose-escalation cohorts included small numbers of patients with varying BL sBCMA levels, making it difficult to discern the role of sBCMA on efficacy for different dose levels. The lowest dose, 16 mg QW, corresponds to the fixed-dose equivalent of the lowest efficacious dose (215 μg/kg) observed in MagnetisMM-1^25^. The virtual patient BL sBCMA levels were calibrated to the available clinical data, such that the proportion of patients with low or high sBCMA levels at BL was comparable to the proportion of patients with the corresponding sBCMA levels in the MagnetisMM-1 and MagnetisMM-3 Cohort A studies. The simulated doses with a weekly dosing frequency were 16 mg, 28 mg, 44 mg, 76 mg, and 152 mg. The weekly dose of 76 mg, or 1000 μg/kg, was proposed as the recommended phase 2 dose (RP2D), and a dose of 1000 μg/kg every 2 weeks (Q2W) achieved a wide PK exposure between that observed for 360 μg/kg QW and 1000 μg/kg QW^25^, but the efficacy of Q2W dose frequency from the start of treatment remained a question of interest. To address this, we simulated an additional schedule of 76 mg Q2W to explore how it compared with 44 mg or 76 mg QW. Lastly, we explored the regimen of 152 mg QW to evaluate the added benefit of a dose higher than the RP2D.

**Fig. 3:**
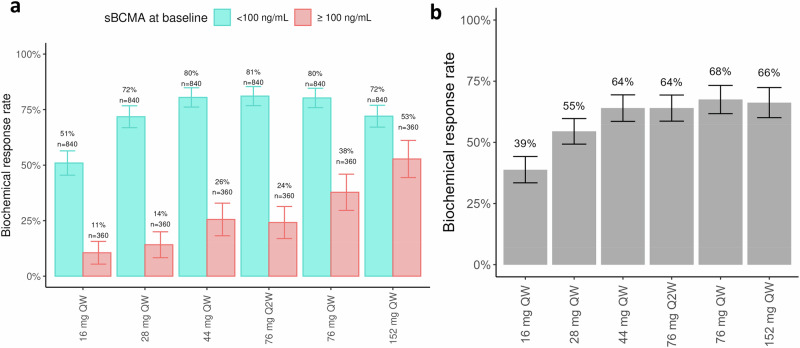
Simulated BRR of ten VPops across increasing dose schedules. Explored doses ranged from 16 mg to 152 mg QW. A 76 mg Q2W regimen was also simulated and resulted in very similar efficacy as measured by the BRR of 44 mg QW. Error bars represent 95% confidence intervals. **a** BRR stratified by baseline sBCMA levels. The model suggests that the optimal regimen for patients with low sBCMA levels (blue) and high sBCMA levels (red) is 76 mg Q2W (BRR, 81%) and 152 mg QW (BRR, 53%), respectively. **b** For all virtual patients, simulated BRR ranged from 39% to 68%, and there was no significant gain in efficacy from 76 mg QW (68%) to 152 mg QW (66%). BRR biochemical response rate, QW once weekly, Q2W every 2 weeks, sBCMA soluble B-cell maturation antigen, VPop virtual population.

For each dose regimen and BL sBCMA subgroup, we calculated the BRR to compare simulated efficacy from the QSP model across evaluated regimens, shown in Fig. 3a. Virtual patients with low sBCMA levels at BL (70% of VPop) showed higher BRR than patients with high sBCMA levels at BL (30% of VPop) for regimens from 16 mg QW to 76 mg QW. Based on these simulations, the 76 mg QW showed the highest efficacy for patients with low sBCMA levels, corresponding to a BRR of 80% in our simulations, which was similar to the BRR observed at 44 mg QW and 76 mg Q2W (~80%) and higher than that at 152 mg QW (72%). For patients with high sBCMA levels, efficacy peaked at 152 mg QW with a BRR of 53%, compared to the 76 mg QW regimen that resulted in a BRR of 38%; both regimens resulted in higher BRRs than lower doses of 44 mg QW and 76 mg Q2W (~25%) (see Fig. 3a). Figure 3b shows the total BRR of the same schedules across all virtual patients (i.e., pooled high and low sBCMA levels). The regimen of 76 mg QW resulted in the highest BRR across all virtual patients (68%) (see Fig. 3b). Considering these simulations, clinical evaluation of the 152 mg QW regimen was deemed unnecessary as it is unlikely to provide additional clinical benefit vs the 76 mg QW regimen for the overall population. The subset of virtual patients with high BL sBCMA levels still achieves clinically meaningful benefit with elranatamab 76 mg QW.

### QSP model simulations of dimer and trimer formation can enhance mechanistic rationale for regimen optimization through dose reduction after initial response

**Fig. 4:**
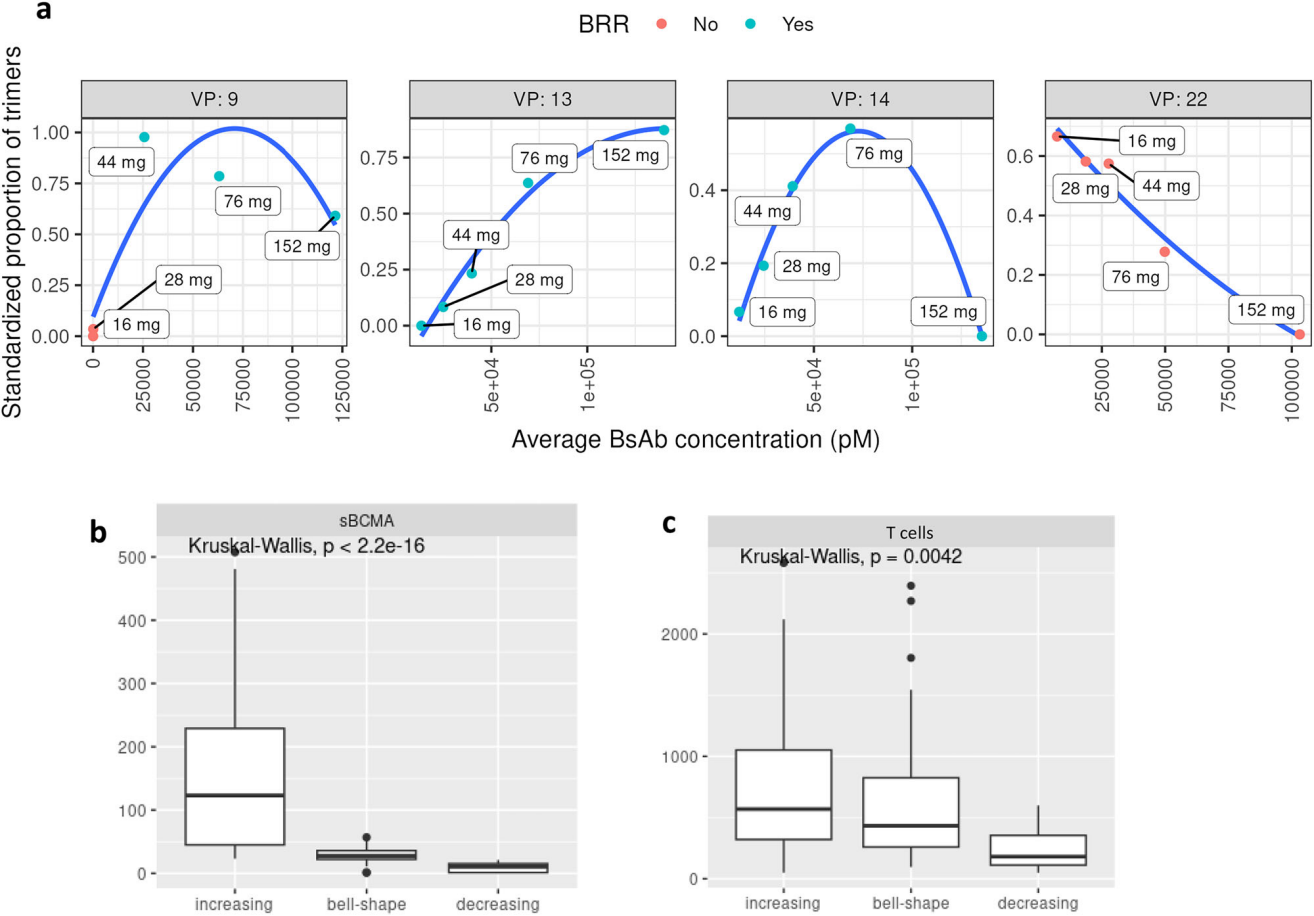
Optimal dose for tumor-killing activity depends on patient-specific and time-dependent factors. **a** The proportion of trimers, or a trimer:BCMA ratio, is plotted against the average BsAb concentration for 4 VPs, with each dose colored by response (red dot: no response, blue dot: biochemical response). A higher proportion of trimer suggests more antitumor activity based on the bell-shaped dose-response theory for BsAbs. VPs 9, 13, and 14 were objective biochemical responders and showed a peak of antitumor activity for lower doses, and a bell-shaped curve is evident for VPs 9 and 14. For these 3 patients, a higher BsAb concentration, a consequence from either a higher dose or lack of dose reduction, could result in inferior efficacy. VP 22, which was a nonresponder at all doses, has a monotonically increasing response curve, suggesting that an optimal dose was not seen within the range of doses simulated. All shown patients have a BL sBCMA < 100 ng/mL for simplicity. **b** sBCMA and **c** T cells in bone marrow were the most significant predictors for dose response curve shape, suggesting that patients with higher counts of these variables would benefit of higher dosing or drug exposure to improve antitumor efficacy. BsAb, bispecific antibody; QW, once weekly; sBCMA, soluble B-cell maturation antigen; VP, virtual patient.

We developed a metric to enable assessment of drug efficacy in our model corresponding to the “effective binding ratio,” which is defined as the number of trimers divided by the number of all BCMA receptors (see Equation 39 in Supplementary Materials). Our computed metric is similar to an effective receptor occupancy metric used in Abrams et al. ^21^ This computed effective binding ratio measures antitumor efficacy at distinct doses for each virtual patient. A high effective binding ratio might indicate optimal antitumor efficacy in alignment with a bell-shaped dose-response curve for BsAbs^45^. More in general, a bell-shaped concentration-response relationship is a well-described phenomenon for ternary complexes^46^. A low effective binding ratio suggests two scenarios: (1) a small amount of drug available that does not build enough trimers for antitumor efficacy or (2) a large amount of drug has saturated the majority of cell receptors (BCMA and CD3), so there is an excess of dimers, which hinders the formation of trimers. We simulated five different doses that were administered weekly for each virtual patient, and we calculated average drug concentration and average effective binding ratio in the first three cycles of therapy. Fig. 4a shows these two metrics for four selected virtual patients with different concentration-response curves. The first and third panels show virtual patients 9 and 14, whose optimal efficacy binding ratios are achieved at drug concentrations corresponding to 76 mg QW, indicating no added benefit from higher doses. In contrast, virtual patient 13, who responded biochemically to 16 mg QW, shows a dose-dependent increase in trimer to BCMA ratio, suggesting potential for deeper response at higher doses. Virtual patient 22, despite having <100 ng/mL BL sBCMA, did not respond at any dose due to limited CD3 receptors, placing them on the declining side of the bell-shaped curve. Overall, while optimal binding ratios vary by individual, simulations support 76 mg QW as the lowest clinical dose providing broad coverage across the virtual population (see Fig. 3b).

To further investigate the determinants of response across doses, we calculated and analyzed the binding ratio versus average BsAb concentration for all virtual patients and catalogued them as illustrated in Fig. 4a and Supplementary Fig. 10b. Based on the shape of the curves, we identified three distinct response patterns: increasing, bell-shaped, and decreasing (Supplementary Fig. 10a). Virtual patients with increasing dose-binding ratio curves are predicted to benefit from higher dosing or drug exposure, whereas those with bell-shaped or decreasing curves may experience reduced efficacy at higher doses (see Supplementary Fig. 10c for BRR trends across groups).

To explore the underlying drivers of these patterns, we stratified virtual patients by dose-binding ratio-curve shape and examined the distributions of model parameters and initial state variables within each group. Using Kruskal-Wallis tests, we identified the most predictive features to be free BL sBCMA and T cell concentration in the bone marrow (Fig. 4b, c). When these variables were assessed jointly, their association with dose-binding ratio shape remained statistically significant (likelihood ratio test, *P* = 0.0023), and, as expected, they were not correlated (Supplementary Fig. 11a). Additional variables evaluated in this analysis are shown in Supplementary Fig. 11b.

**Fig. 5:**
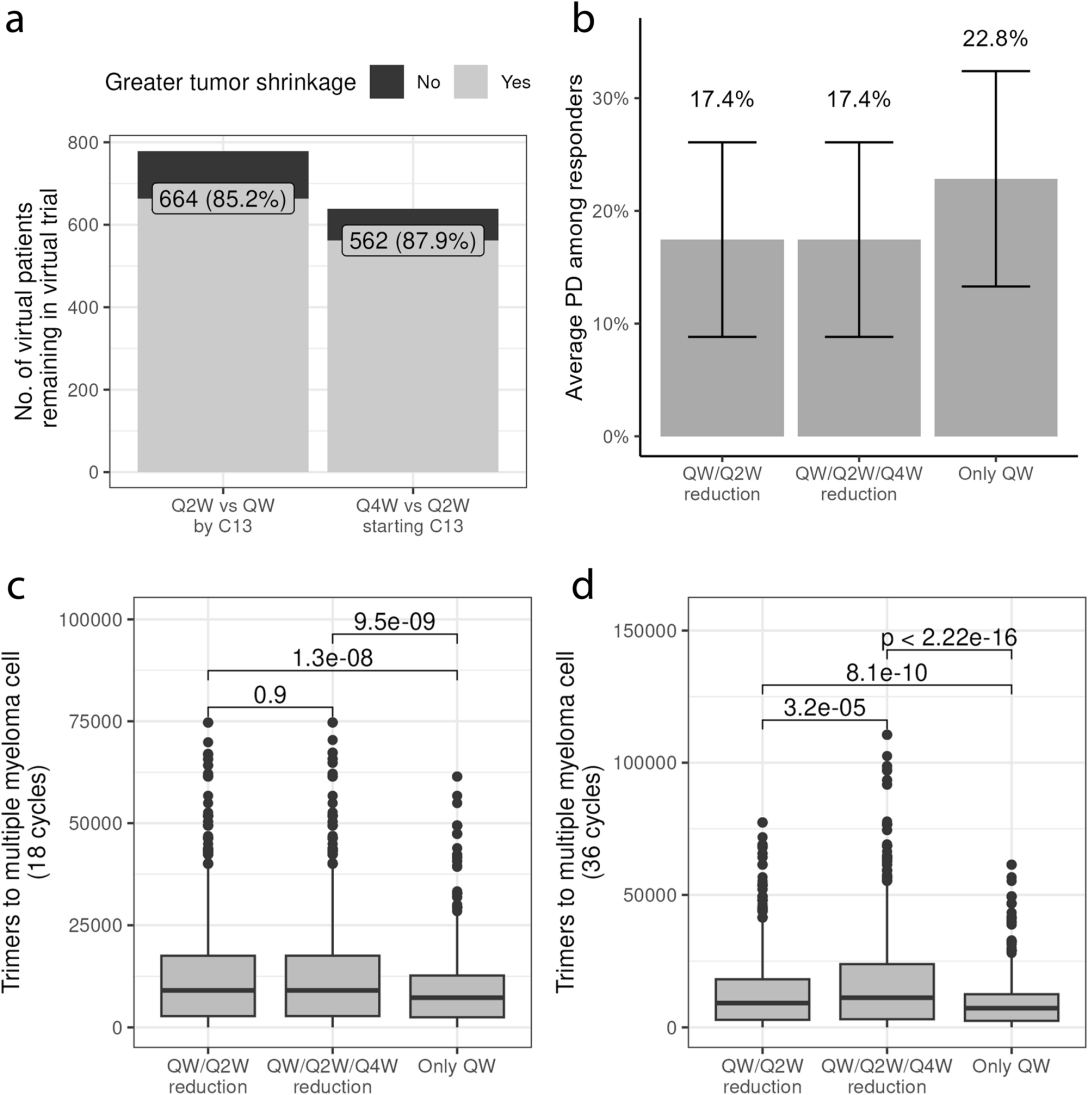
Evaluation of maintenance of efficacy for different dose-reduction regimens. Across the 10 fitted VPops, there were a total of 779 virtual patients that responded to the 76 mg QW regimen with a two–step-up priming. After confirmed biochemical response, we simulated 3 scenarios: (1) switching from QW to Q2W dosing after week 24 of therapy, (2) further switching from Q2W to Q4W starting at cycle 13 (C13), and (3) maintaining QW dosing. **a** A comparison between these regimens showed that 664 of 779 (85.2%) virtual patients saw greater tumor shrinkage with Q2W vs QW dosing following a confirmed response by C13. Starting at C13, when the Q2W to Q4W switch started, among the remaining responders, 87.9% had more tumor shrinkage under the Q4W vs Q2W regimen until the end of therapy. **b** Among the 779 total responders from the 10 VPops, an average of 17.4% of patients progressed after switching to Q2W starting at C7, the same number of patients progressed in the scenario with two dose reductions (Q4W after C12), and 22.8% of responders progressed when they continued with the QW regimen (error bars represent 95% CIs). **c** Average trimer:tumor cell ratio in virtual patients receiving a regimen of QW to Q2W switch vs QW to Q2W to Q4W vs no switch after C18 and **d** C36. On average, there was a statistically significant higher trimer:tumor cell ratio in the regimens with dose reductions, suggesting greater antitumor activity following a dose reduction. All pairwise comparisons were performed using Wilcoxon two-sided paired tests. C cycle, QW once weekly, Q2W every 2 weeks, Q4W every 4 weeks, VPop virtual population.

Besides the total amount of antibody impacting trimers, the dosing frequency can also affect trimer and dimer formation in non-intuitive ways. To this end, we used the model to evaluate the maintenance of response in scenarios when the dose regimen is reduced from QW to Q2W to Q4W after initial response (i.e., from once a week to every other week to once a month dosing regimens). This dose reduction has now been implemented in the clinic for persistent responders, or patients who show a confirmed objective response after six cycles of QW therapy^26^. To facilitate the evaluation of maintenance of response, we computed a simulated progressive disease (PD) event that was derived from International Myeloma Working Group (IMWG) progression criteria. To translate our findings into clinical meaningful outcomes, we defined simulated PD among persistent responders as a 25% increase from the lowest value of an integrated biomarker of a tumor assessment^36^. We set our analysis to only virtual persistent responders because they were candidates for switching to Q2W starting on treatment cycle 7 (C7, each cycle consists 28 days of therapy), so we were able to track their tumor dynamics under a scenario in which they did not switch (continued receiving 76 mg QW after response was confirmed), and one in which they did switch to Q2W (switch to 76 mg Q2W starting at cycle 7 after response was confirmed). Virtual patients that are persistent responders may switch to Q2W at different times starting at cycle 7, since the dose reduction depends on the individual patient’s response. Most virtual patients achieved a confirmed response by cycle 7 while receiving 76 mg QW, with an initial step-up priming regimen, with the median of virtual patients showing almost a clearance in integrated paraproteins by day 100 of therapy, as shown by the red solid line in Fig. 2c. Thus, most virtual patients that were persistent responders transitioned from QW to Q2W starting at cycle 7, as consistent with observed data in MagnetisMM-3^26^. We observed that there was a greater tumor shrinkage in 85.2% of virtual patients with persistent response after a dose reduction (QW to Q2W) compared with a regimen constant dose at QW by cycle 13, as shown in Fig. 5a. A similar analysis showed that in a Q2W to Q4W transition starting at cycle 13, 87.2% of persistent responders remaining in the trial would have greater tumor reduction with Q4W dosing compared with Q2W dosing, also shown in Fig. 5a, b shows that only 17.4% of persistent responders who switched to Q2W showed PD between the time that they switched until day 1095 (3 years) of treatment. The scenario with dose reduction to Q4W after cycle 12 showed a similar result. Under the scenario of no switching, 22.8% of persistent responders showed PD over a period of 3 years of treatment. These simulations suggest that less drug among persistent responders is not expected to be detrimental to maintenance of response and can even result in a longer or better maintenance of response. Our model showed that the average number of trimers to tumor cells significantly increased after the point of dose reduction until 18 cycles of therapy (see Fig. 5c), supporting the evidence of more trimer formation following a dose reduction. The two scenarios with dose reductions showed similar trimer:tumor cell ratios at 18 cycles of therapy, but differences became significant following a total of 36 cycles of therapy (*P* < 0.001 by Wilcoxon test). However, the differences between the QW/Q2W and QW/Q2W/Q4W regimens were considered minimal due to the equal simulated PD.

We took a closer look at the time dynamics of drug trimers to further investigate why less frequent dosing could help increase efficacy (see Supplement). Our model suggests that a switch from QW to Q2W will decrease the ratio of drug-CD3 dimers to T-cell concentrations, thereby increasing availability of CD3 receptors, as shown in Supplementary Fig. 5a. In Supplementary Fig. 5b, we show an increase in the trimer:tumor cell ratio after the Q2W switch, suggesting that trimer numbers were increasing under Q2W compared with QW dosing, thus increasing tumor-killing activity. There was little difference in the ratios of BsAb-BCMA dimer:tumor cell between the dosing schedules, as shown in Supplementary Fig. 5c, suggesting that most BCMA receptors were engaged with BsAb in a dimer complex. Thus, under a Q2W schedule when tumor burden is sufficiently low, BCMA-BsAb dimers are more likely to find an available CD3 receptor to bind and form a trimer, leading to better maintenance of response.

### Evaluation of response criteria for the QW to Q2W transition

**Fig. 6:**
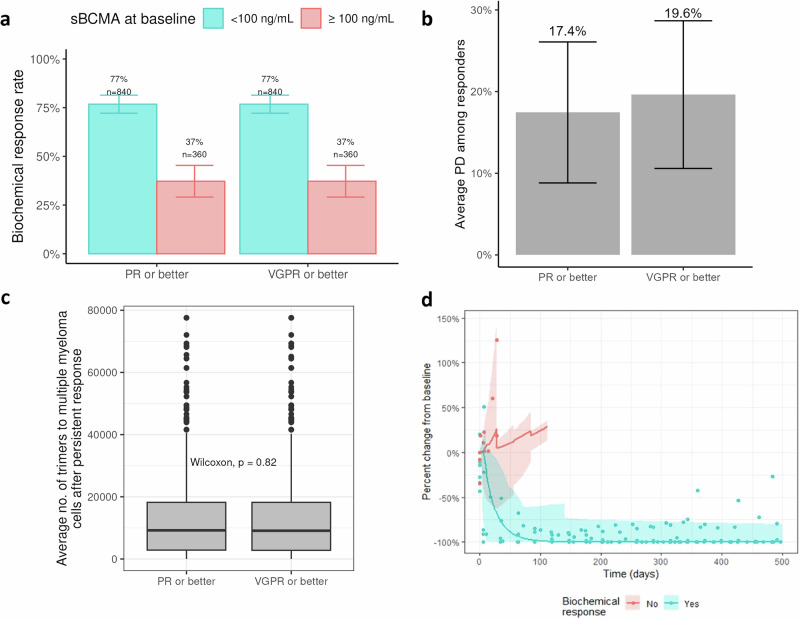
Stricter response criteria for QW to Q2W transition may decrease maintenance of response in few patients. **a** Biochemical response, defined as confirmed PR or better, is equal between both regimens, with 77% and 37% of virtual patients with low and high sBCMA levels at baseline, respectively, reaching a biochemical response. Error bars represent 95% CIs. Among these responders, **b** the predicted PD percentage of the scenarios of switching from QW to Q2W for PR or better vs VGPR or better response suggest that PR or better response for switching can be beneficial. **c** Box plots of trimer:tumor cell ratio across virtual patients are not significantly different between the two scenarios. **d** Simulation of median levels of paraproteins under a regimen of 76 mg Q2W is shown in solid curves with 95% CIs, and data from 13 patients with RRMM are shown in dots, colored by biochemical response. PD progressive disease, QW once weekly, Q2W every 2 weeks, RRMM relapsed or responsive multiple myeloma, sBCMA soluble B-cell maturation antigen, VGPR very good partial response.

Next we investigated whether a more stringent criterion of a deeper simulated clinical response to transition to Q2W would influence the maintenance of efficacy, thus effectively testing the robustness of the model prediction with respect to clinical switching criteria. As outlined above, a dose reduction from QW to Q2W starting on C7 happens once a patient reaches partial response (PR) or better (see Methods for criteria and calculation of response). To investigate an alternative threshold for switching from QW to Q2W dosing, we tested another criterion of a very good partial response (VGPR) or better (i.e., ≥90% decrease in paraprotein levels from BL)^25,37^. We compared this scenario requiring VGPR or better to switch with the one that requires PR or better after cycle 6. In both scenarios, a two–step-up priming regimen was simulated (i.e., 12 mg on cycle 1 day 1, 32 mg on cycle 1 day 4, and 76 mg on cycle 1 day 8 with QW thereafter until eligible for transition to Q2W). As expected, the BRR did not change between the two scenarios since a biochemical response in our algorithm is defined as PR or better, which is reached before a VGPR level is reached (see Fig. 6a). Maintenance of response analysis, in Fig. 6b, shows that transitioning to Q2W following a PR or better results in fewer PD events than transitioning to Q2W following a VGPR or better criterion (17.4% vs 19.6% PD among responders, respectively). In other words, the QW to Q2W transition criterion of achieving VGPR or better had 17 additional PD events (2.18% of persistent responders) compared with the reference (recommended) scenario. This finding suggests that earlier dose reductions might be better, contrary to the perception of waiting for a deeper response to reduce the dose. Despite seeing a better maintenance of response when transitioning to Q2W after PR or better, there was no statistically significant difference in the number of trimers per tumor cell between scenarios, suggesting very little difference in tumor-killing optimization between schedules across all virtual patients (see Fig. 6c).

### Evaluation of model prediction performance for additional regimens and time-varying biomarkers

To further evaluate the robustness of our VPop calibration framework, we explored various simulated outputs not used during model calibration and compared them with observed data. First, we compared free sBCMA and drug concentrations with the simulated values in the central compartment of one VPop, as shown in Supplementary Fig. 7. The 95% CIs of the free sBCMA simulations captured the observed median of the MagnetisMM-3 study, while the free drug concentration was initially overestimated and then underestimated after day 50 of treatment. To examine whether our model accurately described IL-6 trends, we used the cohort arm that received the RP2D in the MagnetisMM-1 study to tune model IL-6 peaks. This cohort followed a one-step priming regimen: 44 mg on cycle 1 day 1 and 76 mg on cycle 1 day 8 and weekly thereafter. We aimed to have median projections match median observations. Supplementary Fig. 8 shows the model predictions but following a two-step priming regimen: 12 mg cycle 1 day 1, 32 mg cycle 1 day 4, 76 mg cycle 1 day 8, and weekly thereafter. Compared with the observed IL-6 levels from the MagnetisMM-3 Cohort A study, the median of the simulated IL-6 peaks matched the data median, especially during the priming doses, but variability of observed IL-6 levels was underpredicted.

To validate our model, we tested the predictions against another arm within the MagnetisMM-1 study. In the MagnetisMM-1 Q2W priming arm, participants with RRMM (*n* = 13) received 44 mg on cycle 1 day 1 and 76 mg on cycle 1 day 8 and every 2 weeks thereafter. The enrollment criteria for this arm were the same as those from the MagnetisMM-1 QW and MagnetisMM-3 Cohort A studies. We simulated this dosing regimen using one of the ten fitted VPops that was chosen randomly. Fig. 6d shows the paraprotein dynamics of MagnetisMM-1 Q2W participants compared with the simulated dynamics of a VPop, stratified by biochemical response. The 95% prediction interval of our model covers most of the observed percent changes from BL. Our model accurately predicted the year long-term dynamics for patients who responded to treatment under a reduced dosing interval, and it was able to predict the quick progression of disease as measured by the increase of paraprotein levels among those that did not reach an objective biochemical response.

## Discussion

In this work, we outlined the development and calibration of a QSP model that incorporates the mechanism of action of elranatamab in an RRMM patient population and used the model to elucidate the mechanistic sources that may underpin distinct efficacy outcomes. This work provides mechanistic insights on the potential benefits of reducing the dosing frequency for sustained maintenance of responses, a phenomenon intricately tied to the complexity of receptor binding and the interplay between T cells and myeloma cells. Furthermore, through the simulation of VPops, it is possible to systematically evaluate mechanistic sources contributing to the heterogeneity observed in treatment responses. This model framework was used to inform dosing decisions in currently ongoing studies of patients with RRMM, thereby enhancing the precision and effectiveness of therapeutic interventions in this context.

The QSP model has core components established by prior published mechanistic models^18,45,47^, as well as novel components from model calibration with available data. The model described the dynamic changes in MM cells over time in a tumor site of action compartment, while drug binding dynamics and transport through compartments were refined to elranatamab. A VPop approach was used to explore sources of variability and uncertainty, and model calibration relied on early clinical human data^25,26^. The use of human data for our analysis is an important feature since it strengthens the QSP model by making it less prone to bias^48^ (i.e., modeling the same compound and patient population as that of the data used for calibration) and accurately replicating the observed distribution of efficacy endpoints, as well as other biomarkers.

The QSP model was informative in evaluating the varying mechanistic components that contribute to a bell-shaped dose-response curve inherent to BsAb therapies^45,46,49^. Saturation of binding to one or both targets within the tumor environment results in a nonlinear relationship between dose and trimer to BCMA ratio, which can give rise to a nonlinear dose-response curve. Individual simulations demonstrated the variability in the relationship between the dose/exposure and maximal trimer formation. This finding suggests that each patient has their own bell-shaped curve. However, among the doses explored with our model, 76 mg QW was the lowest dose that provided the maximal benefit (i.e., BRR) for the overall patient population; thus, our model supports the approved dose.

Higher sBCMA levels have been associated with poorer outcomes^43,50,51^, which may be due to its correlation with clinically relevant covariates (e.g., tumor burden and staging) or through its impact as a drug sink. Our findings are consistent with data reported from other approved BsAbs for myeloma, teclistamab and talquetamab, which reported higher distribution of baseline sBCMA among nonresponders vs responders^44^. Similar findings were noted for the BCMA-targeting antibody-drug conjugate belantamab mafodotin^52^. Thus, sBCMA has been shown to be a prognostic biomarker for response, with higher levels associated with lower responses in all BCMA-targeting BsAbs to our knowledge. We integrated the conclusions derived from these analyses by fitting our objective function to different sub-populations after specifying their sBCMA levels at BL. As a result, our model was able to inform dosing for patients with different levels of sBCMA. Although the BRR in virtual patients with high sBCMA levels was higher at 152 mg QW compared with 76 mg QW, an opposite trend was noticed among patients with low sBCMA levels. Additionally, patients with high sBCMA levels were shown to have other poor prognostic factors (i.e., higher tumor burden, baseline plasma cells, EMD status) that may be addressed in the future via combinations of elranatamab with other antimyeloma agents^53^. Model results should be interpreted in the context of elranatamab biochemical response in RRMM patients who were triple-class refractory and BCMA treatment naïve. Caution should be exercised in extrapolating our results for the 100 ng/mL of BL sBCMA as a cut-off to influence treatment outcome in other MM patient populations or with other BCMA-targeting treatments. Given the minimal impact on the BRR for the overall population and the potential detrimental impact for BRR on patients with low sBCMA levels who represent the larger portion of the targeted population, our analysis supported 76 mg QW as the recommended full treatment dose.

Key findings from our analysis indicate that BL sBCMA and T cell levels in the bone marrow are critical determinants of dose-response behavior. Patients with higher BL sBCMA require more drug to overcome the drug sink and generate sufficient trimers for anti-tumor activity. Similarly, higher T cell levels enhance trimer formation and efficacy with increasing dose. When both factors are limited, saturating effects emerge, resulting in bell-shaped or decreasing dose-response curves.

A key finding of this work is that persistent responders would benefit from gradual reduction of the dosing frequency from QW to Q2W. The mechanistic rationale to this somewhat counterintuitive result involved decreased receptor availability. Our results suggest that at the beginning of therapy, most patients will benefit from 76 mg QW to reach an objective biochemical response. Upon achieving biochemical response, levels of free BCMA and CD3 receptors decrease, requiring less BsAb in the system to form trimers. If such gradual reduction in the dosing frequency is not applied, the BsAb will saturate the free receptors, creating an excess of dimers in the system and inhibiting the formation of functional trimers that trigger tumor death. With the lower trimer formation, the risk of the tumor-escaping therapy increases, resulting in a higher probability of disease progression.

Our analysis also explored the criterion for initiating a reduction in dosing intensity and whether requiring deeper responses to switch (e.g., VGPR or better) provides additional benefit in maintenance of responses compared with earlier switching requiring only PR or better. Our findings indicated that earlier switching to Q2W at shallower responses (PR or better vs. VGPR or better) was associated with less probability of disease progression. Switching from QW to Q2W has been adopted by two approved BCMA-targeting BsAbs, elranatamab and teclistamab. While the switching criteria are PR or better starting at cycle 7 for elranatamab, deeper responses are required for teclistamab (i.e., complete response or better for a minimum of 6 months)^54,55^. Despite the lack of data on a continued QW dosing regimen, our model simulations are consistent with findings from the MagnetisMM-3 study, in which durable responses were maintained after reducing the dosing interval from QW to Q2W.

The mechanisms and assumptions of our model proved to be sufficient to predict IL-6 dynamics in the MagnetisMM-3 study. The assumptions for the cytokine release model component were modified from a semi-mechanistic approach that addressed the attenuation of cytokine release with repeated dosing^47^. We selected IL-6 since it has been significantly associated with CRS^56,57^. A few published models have used IL-6 projections to recommend dose-fractionation schedules for CRS mitigation^22,58^. While our model has yielded valuable insights with regard to efficacy projections, more work is needed in the future to incorporate more mechanistic details related to key cytokines and immune components that are key drivers of cytokine release. Future versions of this model will focus on addressing these limitations and extending the model to other patient populations or combination settings.

In summary, we have outlined the development and refinement of a mechanistic modeling framework that can connect several layers of immune and tumor dynamic components with a multitude of clinical efficacy biomarkers and summary endpoints from phases 1 and 2 MagnetisMM trials. When paired with a clinically calibrated and validated virtual population workflow, the QSP model outlined in this work can provide important mechanistic rationale for regimen optimization for complex modalities like elranatamab. The QSP modeling framework is flexible enough to permit expansion and further refinement in the future as more data from BCMA-directed BsAb therapy become available for relevant MM patient populations.

## Methods

### Virtual trial simulations

An assessment of model uncertainty and variability was performed through exploration of parameter space using a VPop parameter sampling approach^38,39,59^. At a high level, we first generated a preliminary set of plausible parameter sets and then further filtered the plausible population (PP) into a VPop by evaluating against clinical trial data. The plausible parameter set values/ranges were refined from literature or reference experimental data such that for each parameter, all the corresponding model outputs (see Supplementary Table 2), such as tumor doubling times, fell within acceptable ranges. The model initial conditions for PP were obtained from sampling probability distributions fitted to BL observations of sBCMA, M-protein, and FLC pooled from data collected from patients in the MagnetisMM-1 study and MagnetismMM-3 Cohort A study; we relied on literature values to initialize other variables of the model. We refined the PP through optimization techniques to permit generation of subsets of parameters that constituted VPops that matched observed patients’ paraprotein time courses and computed a serum-based BRR per IMWG criteria^37^. We analyzed multiple VPops to explore variability in simulated variables and predict the efficacy of elranatamab for various simulated doses and regimens of interest.

### Clinical data available for model calibration

In the MagnetisMM-1 study, patients received elranatamab subcutaneously as a single agent^25,60^. Each dose-escalation cohort used in our analysis was composed of three to six patients. The MagnetisMM-3 study, an ongoing multicenter, phase 2 trial, investigated the efficacy and safety of elranatamab in patients with RRMM^26^. We used all patients in Cohort A (patients with no prior exposure to BCMA-targeting agents) for model calibration. Longitudinal biomarkers from blood assays used for analysis from all patients included serum M-protein FLC, sBCMA, cytokines, and drug concentration. Integrated paraprotein was derived as a combination of serum M-protein and serum FLC observations from the study datasets. Separate serum biomarkers could not be used for model calibration for patients that had incomplete data, i.e., M-protein or FLC at baseline that were below the limit of detection or unmeasurable per IMWG criteria^36,37^. In the case in which both measurable serum paraproteins were available, M-protein was prioritized and used only if it was measurable at baseline (≥0.5 g/dL) in alignment with IMWG criteria^36^. In Supplementary Fig. 4, we delineate our algorithm for integrated paraprotein selection developed using IMWG priority, which yields an integrated paraprotein per patient.

### Model calibration

At model initialization, we sampled each of these parameters from a continuous uniform distribution with bounds that were informed from the literature (see Supplementary Fig. 1 and Supplementary Table 1). A few key parameters, such as association and disassociation rate constants of elranatamab to BCMA and CD3 receptors, were fixed at available preclinical assay values; receptor densities were obtained from the literature (see Supplementary Table 3). We used BL internal clinical or published data from similar patient populations to build probability distributions for observed variables in the model (see Supplementary Table 2 for a list of model states). We fitted a log-normal distribution for literature values for T-cell counts in patients with MM^61^. We used the fitted distributions of state variables to sample initial states and perturbed model parameters individually and independently ~10,000 times. A first plausibility check was applied to these trials by selecting only the parameter sets that would yield doubling times of plasma cells and serum M-protein under no treatment that are within extreme values observed in the literature^41,42^.

Based on IMWG criteria, a virtual patient was defined as a simulated biochemical responder if the patient achieved ≥50% decrease from BL in serum integrated paraprotein (see Supplementary Fig. 4 for definition) levels lasting over two treatment cycles^36,37^. The simulated doses in the QSP model for all PPs included all subcutaneous doses in the MagnetisMM-1 study: 80, 215, 360, 600, and 1000 μg/kg administered QW^25^. Additionally, we simulated two regimens with priming: (1) 600 μg/kg on day 1 and 1000 μg/kg on day 8 and thereafter QW to match part 1 and part 1.1 cohorts in the MagnetisMM-1 study^25^ and (2) 12 mg on day 1, 32 mg on day 4, followed by 76 mg on day 8 and thereafter QW to match the regimen in the MagnetisMM-3 study^26^. We removed plausible patients from simulated trials when they met *a* ≥ 25% increase from nadir in simulated serum paraprotein, which is a criterion for PD according to IMWG^36,37^. Each plausible patient was assessed every 3 or 4 weeks of simulated treatment (4 weeks under fractionated dosing, in line with study protocols^25,26^) and removed from the cohort when the patient’s dynamics satisfied the outlined benchmarks of PD. Lastly, we implemented a switch in the dosing frequency at the patient level from QW to Q2W starting at week 25 conditional on a plausible patient showing a confirmed biochemical response by week 25 to match all study protocols.

We stratified both observed and virtual patients by high vs low BL sBCMA levels, defining high BL sBCMA levels to be ≥100 ng/mL and low BL sBCMA levels otherwise, and we estimated BRR for each stratum and study. The sBCMA threshold of 100 ng/mL was chosen by applying a logistic regression iteratively with BRR as output and a binary covariate of sBCMA status (low if below tested threshold and high if at or above tested threshold), and the threshold resulting in the lowest *P* value was selected. The status of low/high sBCMA levels in PPs was used to match observed patients with corresponding status of sBCMA levels during model fitting. From approximately 4000 PPs, we selected ten distinct VPops, each composed of 120 virtual patients, such that each VPop matched clinical studies when simulated under similar treatment conditions using a genetic algorithm (see Supplementary Fig. 3 and Supplementary materials for a description of the optimization algorithm).

### Simulation of various regimens of BsAb with calibrated virtual populations

The regimens explored in the simulations compared efficacy predictions by varying the amounts and/or frequency of dosing. Additionally, we explored reducing dosing frequency after virtual patients achieved biochemical response by cycle 7 of treatment. In other words, we assessed the interim virtual patient response by extracting the serum M-protein or FLC dynamics in the last two cycles and flagging the virtual patient if there was a PR or better based on these biomarkers. If a virtual patient was flagged, the virtual patient was switched to the Q2W regimen. Otherwise, the virtual patient remained on a QW regimen. To evaluate the effect of QW to Q2W transition, we simulated a hypothetical scenario with virtual patients receiving the QW regimen despite response status to compare duration of response between a QW dosing approach with no switch, Q2W, and another in which responders are switched to a Q2W regimen as implemented in the clinic^26^.

## Supplementary information

Supplementary Information

## Data Availability

Upon reasonable request and subject to review, Pfizer will provide the data that support the findings of this article. Subject to certain criteria, conditions, and exceptions, Pfizer may also provide access to the related individual de-identified participant data. See https://www.pfizeroncologydevelopment.com/trials for more information.

## Code availability

The code to reproduce figures in the manuscript will be available in a public Pfizer repository: https://github.com/openPfizer/.
