# Supplementary material for: Leveraging quantitative systems pharmacology modeling for elranatamab regimen optimization in relapsed or refractory multiple myeloma
Source: NPJ Syst Biol Appl. 2025 Sep 1;11:102. doi: 10.1038/s41540-025-00585-z (PMC12402305; doi:10.1038/s41540-025-00585-z)
Supplement: Supplementary file 1 — Supplementary Information [file 41540_2025_585_MOESM1_ESM.pdf]

Supplementary Materials for "*Leveraging quantitative systems pharmacology modeling for elranatamab regimen optimization in relapsed or refractory multiple myeloma* "

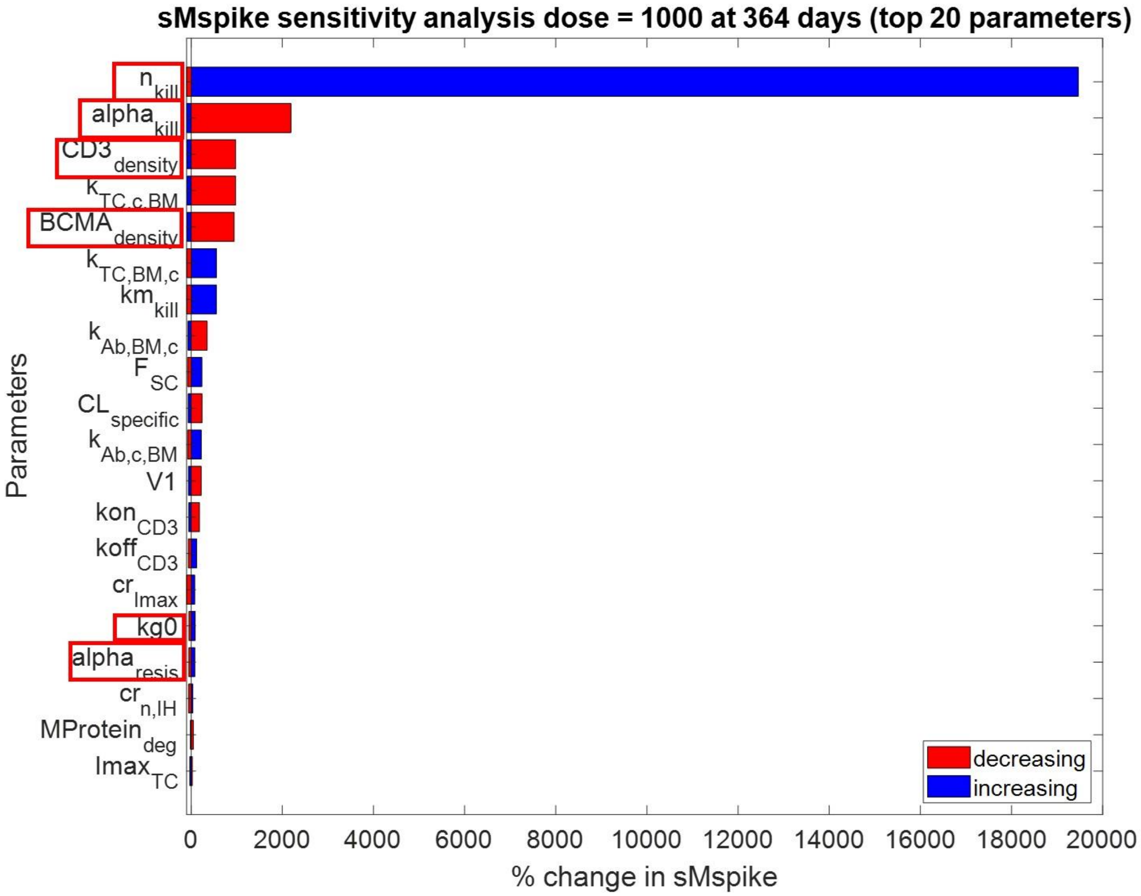

Supplementary Figure 1 Local sensitivity analysis, top 20 parameters shown. Each parameter was decreased and increased by 25% and we measured its effect on serum M-spike at treatment day 364 with dose of 1000 ug/kg. The six parameters boxed in red were selected to vary based on LSA, and on prior knowledge of known heterogeneity or lack of knowledge on reference value used. Parameters not shown in top 20 that are perturbed are: β_{resis}, τ_{MM}, k_{g1}, and they were selected due to their uncertainty. All parameters and their descriptions are listed in Supplementary Table 3.

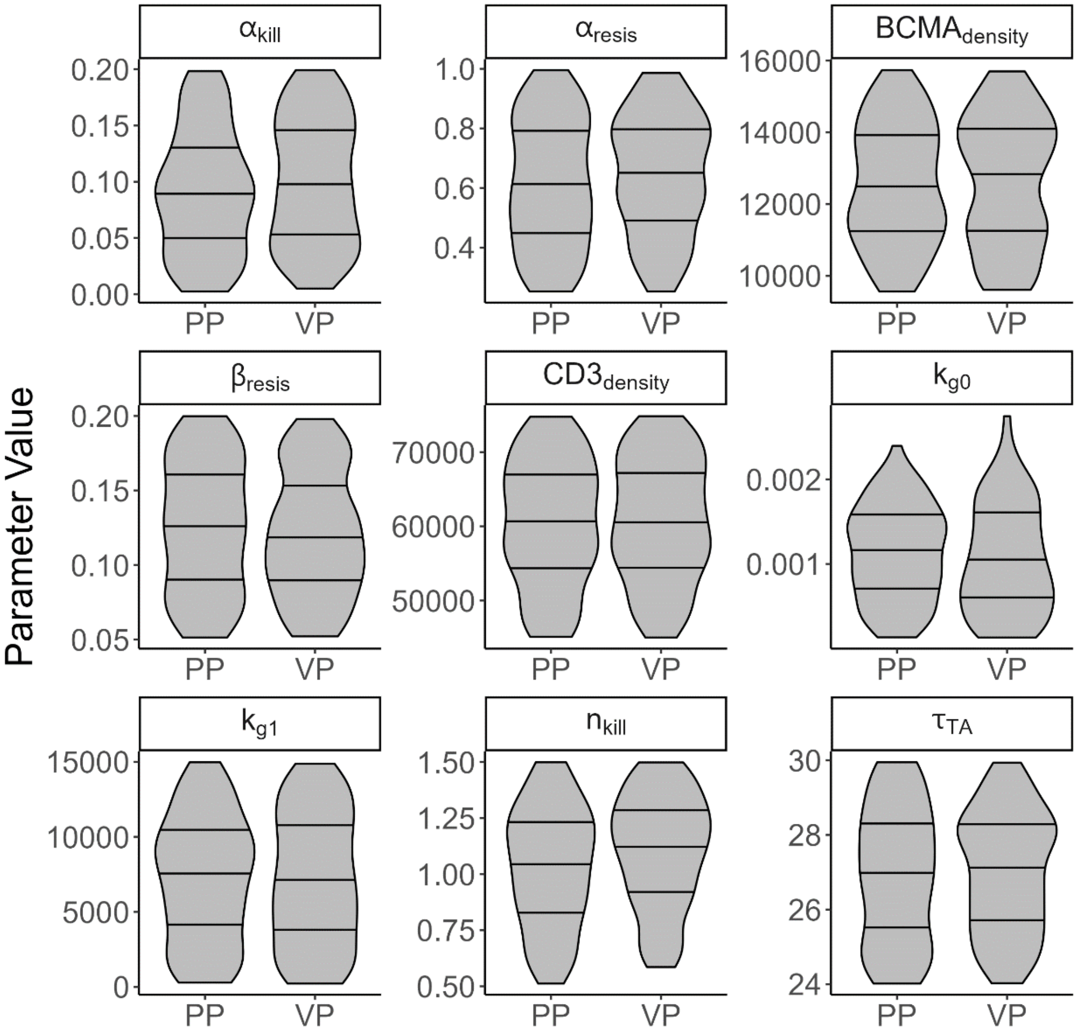

Supplementary Figure 2 Parameter distributions of plausible patients (PP, n > 4000) and virtual patients (VP, n = 120, VP is a subset of PP).

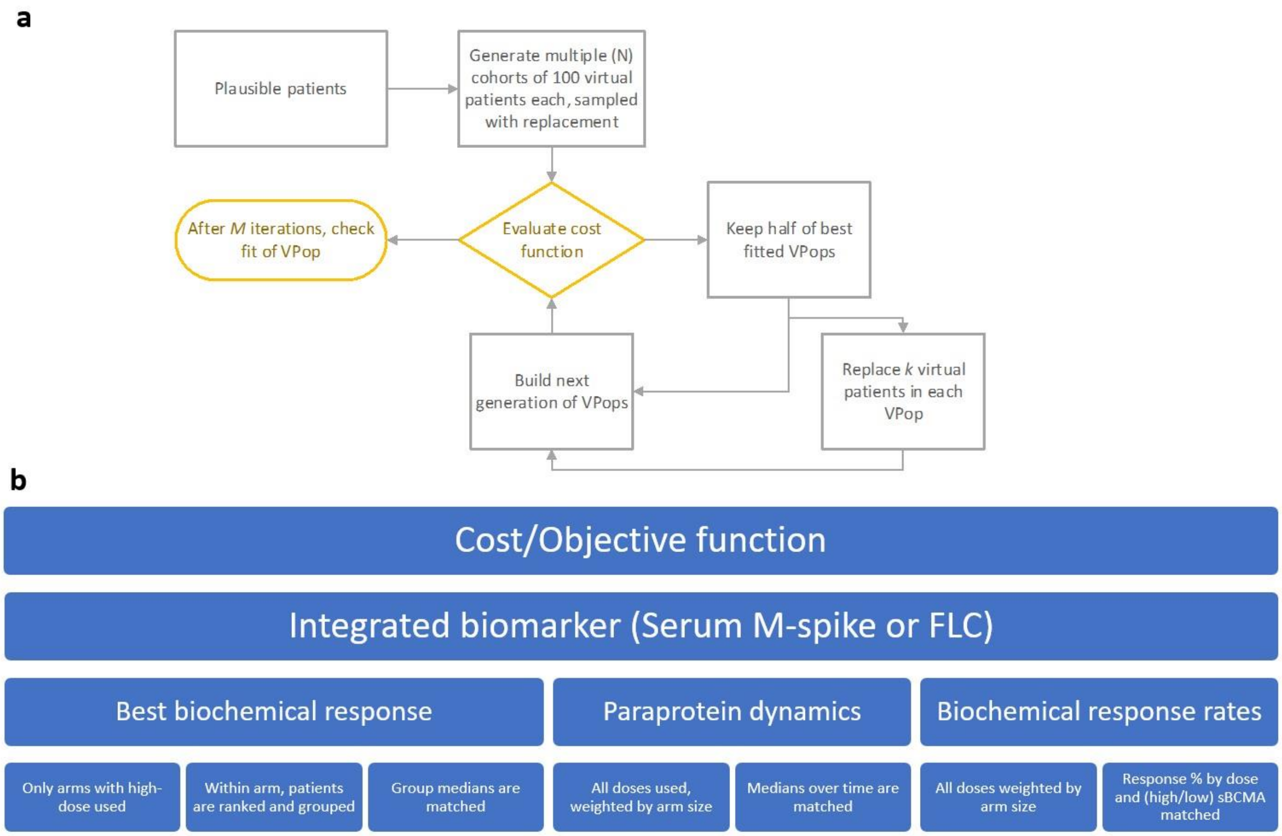

Supplementary Figure 3 Genetic algorithm. (a) Schema of genetic algorithm when selecting virtual patients from plausible patients. The collection of virtual patients is called a virtual population. (b) Optimization function uses 3 metrics that use an integrated biomarker as input. The 3 metrics are best biochemical response (percent change from baseline at best response), paraprotein dynamics (percent change from baseline), and biochemical response rates. We stratified patients' biochemical response by sBCMA levels at baseline (low vs high) by finding the threshold that best separates objective biochemical responders from non-responders using a logistic regression with response as the outcome and high/low sBCMA as a dichotomous predictive variable.

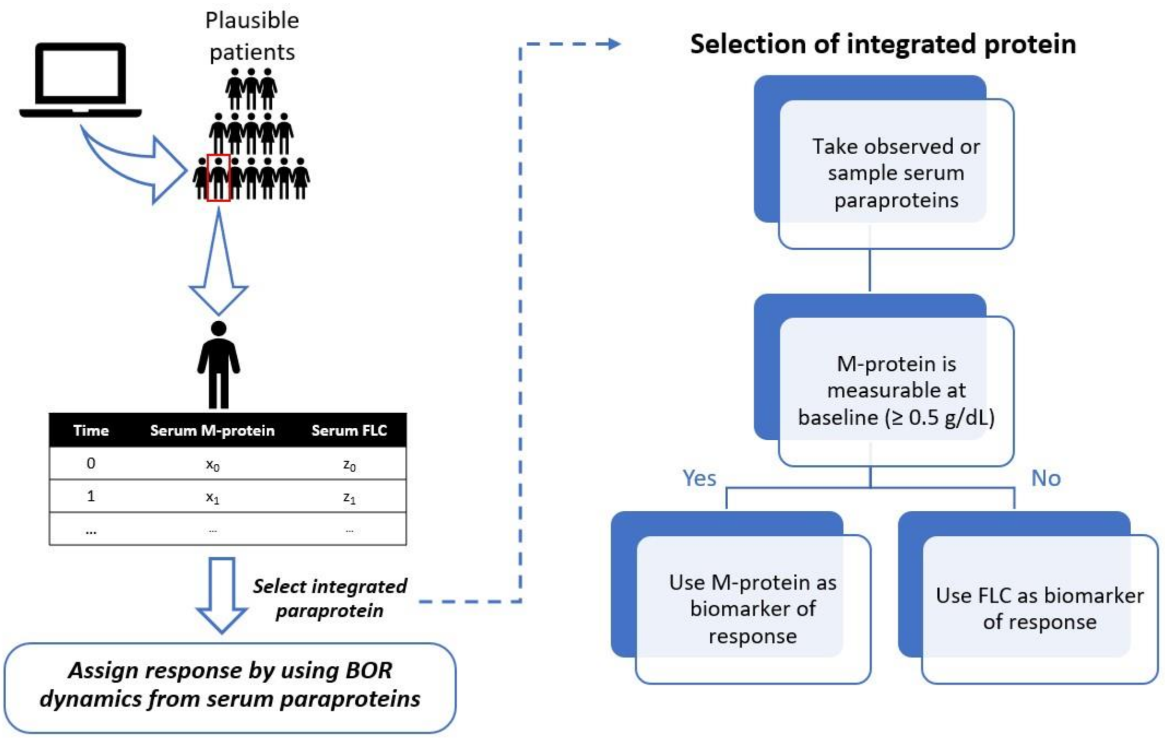

Supplementary Figure 4 Calculating response metrics based depends on selected integrated paraproteins. Each plausible patient will have simulated longitudinal serum M-protein and serum FLC value, but only one paraprotein will be used for response calculation and this paraprotein is referred to as an integrated paraprotein. We select the integrated paraprotein by observing the serum paraproteins at baseline, prioritize M-protein if it is measurable (greater or equal to 0.5 g/dL), otherwise use FLC as biomarker of response. This will result in responses based on two different biomarkers across the plausible patients. The dynamics of the selected integrated paraprotein is then used to calculate response metrics.

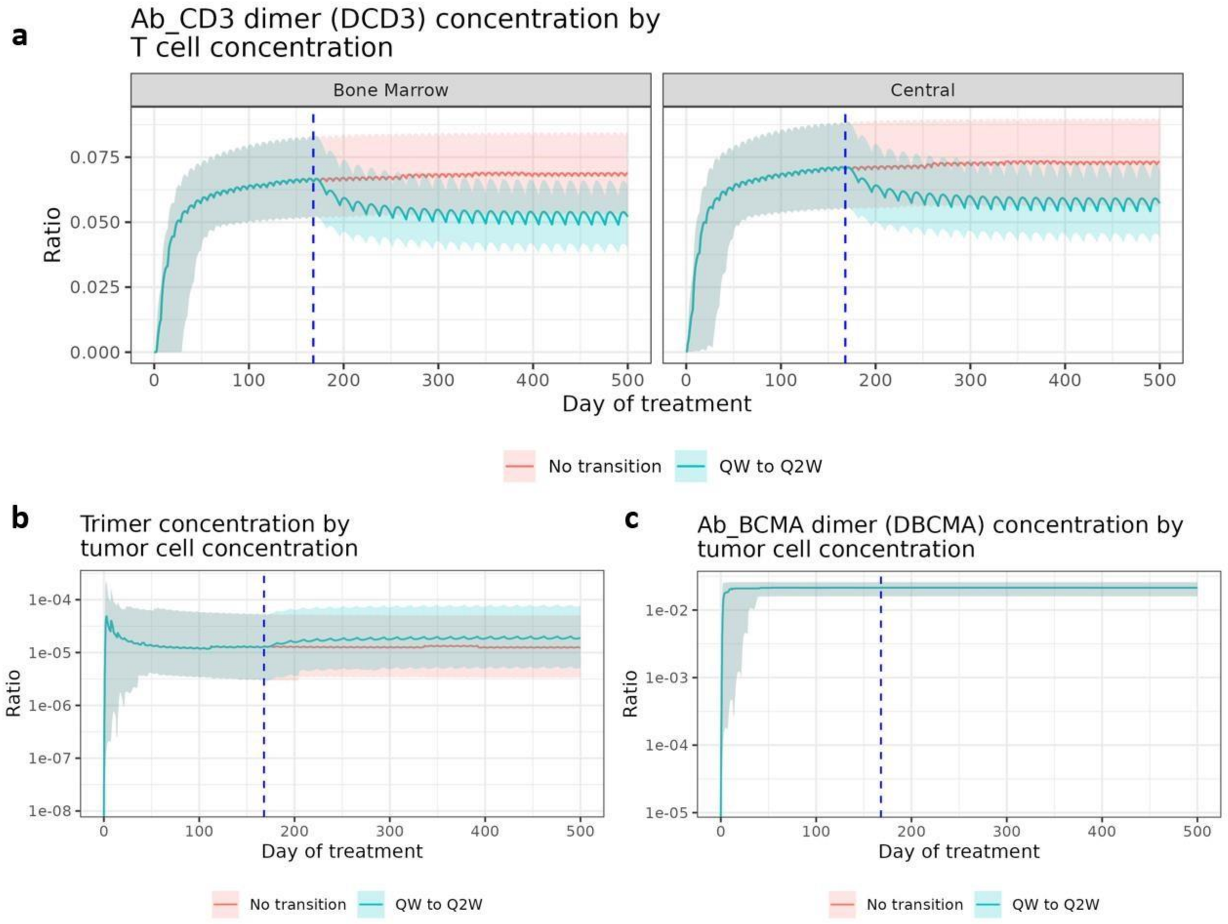

Supplementary Figure 5 Dimers per cell ratios to analyze dose-reduction. (a) After a QW to Q2W switch, the ratio of drug-CD3 dimers standardized by T cells in each compartment decrease, compared to a constant QW regimen, shown in blue and red curves, respectively. The decrease of dimers relative to T cell concentrations suggests that availability of receptors will increase and be able to bind and create trimers. (b) Trimer production, relative to tumor burden, is increased after a QW to Q2W switch, suggesting greater antitumor activity. (c) Drug-BCMA dimers relative to tumor burden in the bone marrow remain roughly similar between the schedules. In all figures, the median ratio is shown in a solid curve and the 95% confidence interval is shown in shaded areas.

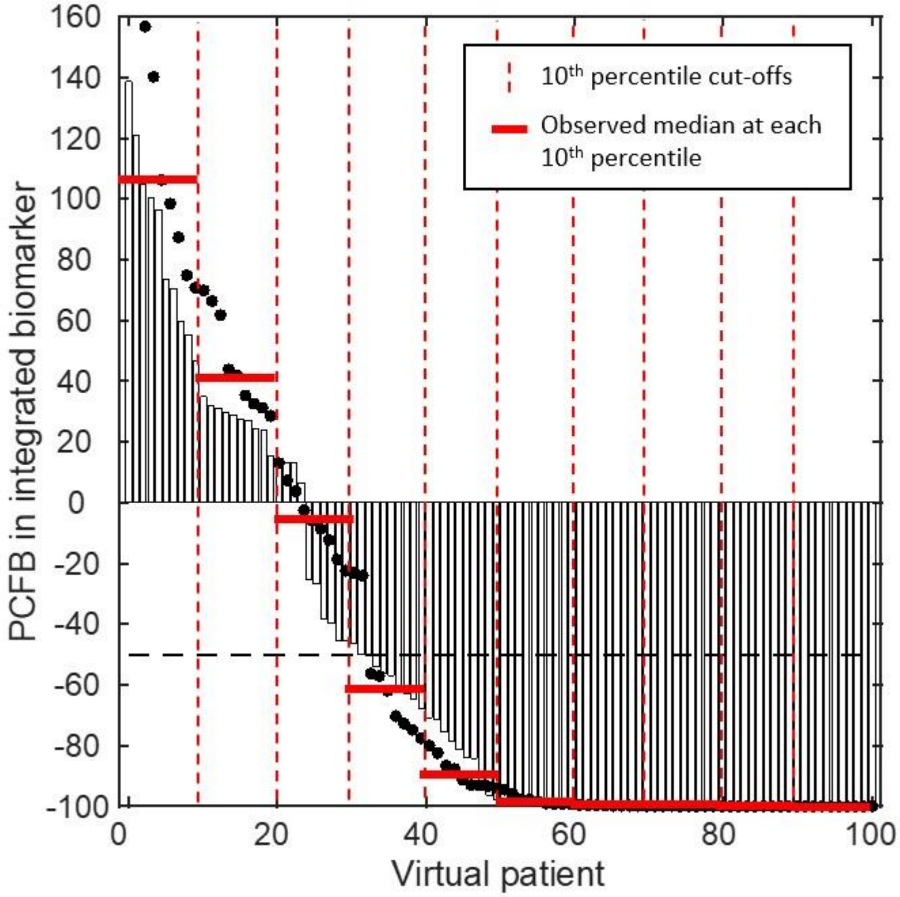

Supplementary Figure 6 Calibration of best biochemical response (BBR) or waterfall plots to the MM-3 cohort A study. BBRs from clinical data are ranked in descending order, then they are grouped into 10 groups (shown vertical dashed red lines). For each group, the median BBR is estimated (shown in vertical solid red lines). Our optimization algorithm minimizes each group's median BRR between the virtual patients and the clinical data. PCFP: Percent change from baseline.

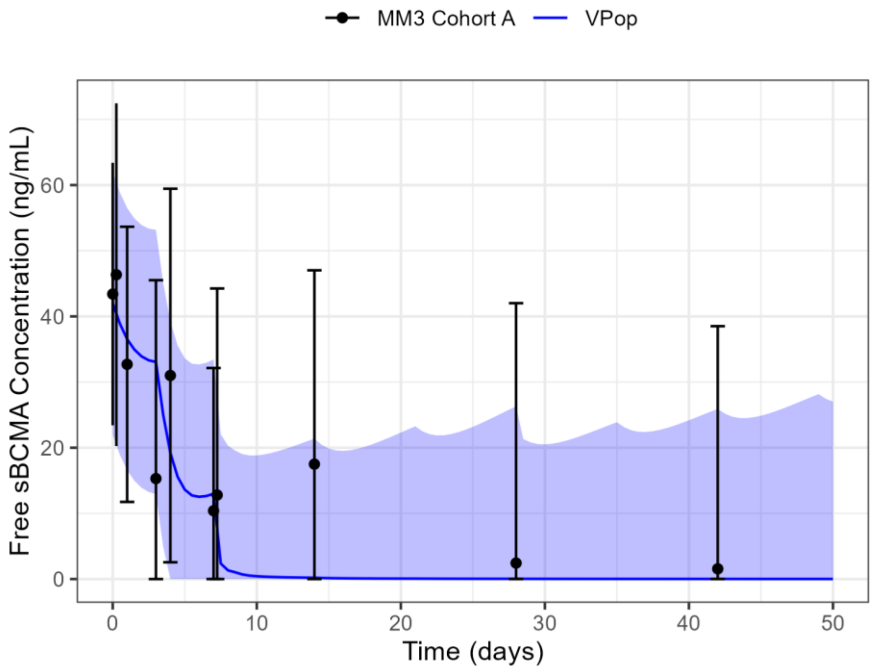

Supplementary Figure 7 Timeseries of free sBCMA in central compartment. Median and 95% confidence interval of simulations are shown in solid lines and grey shaded areas, respectively. Median and 95% confidence interval of MM-3 cohort A study are shown in black dots and black error bars, respectively. In the data, these variables correspond to biomarkers measured from two distinct assays. The simulated regimen is 12 mg on C1D1, 32 mg on C1D4, 76 mg on C1D8 and QW thereafter until C6, then 76 mg Q2W (if confirmed response) for 6 additional cycles, then 76 mg Q4W (if response is maintained).

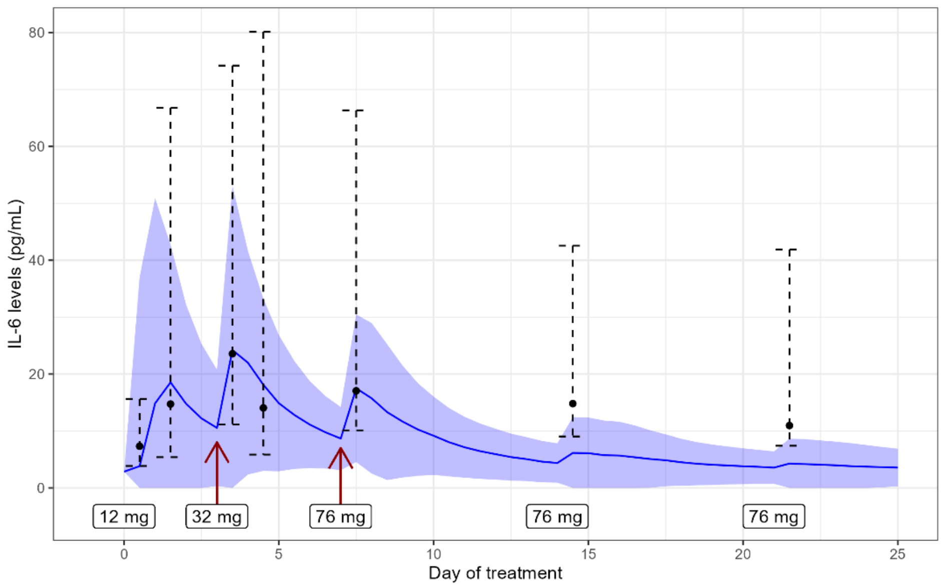

Supplementary Figure 8 Cytokine dynamics in QSP model include attenuation of peaks and replicate observed median of IL-6 levels in MM-3 study. Visual check shows that fitted VPops accurately follow observed trends of IL-6 for MM3 study, which followed a priming regimen of 12 mg on C1D1, 32 mg C1D3, and 76 mg C1D8 and weekly thereafter. Median and 95% confidence intervals of observed IL6 levels in MM3 patients are shown in black dots and black error bars, respectively. Median and 95% prediction interval of projected IL-6 levels are shown in a blue solid line and filled area, respectively.

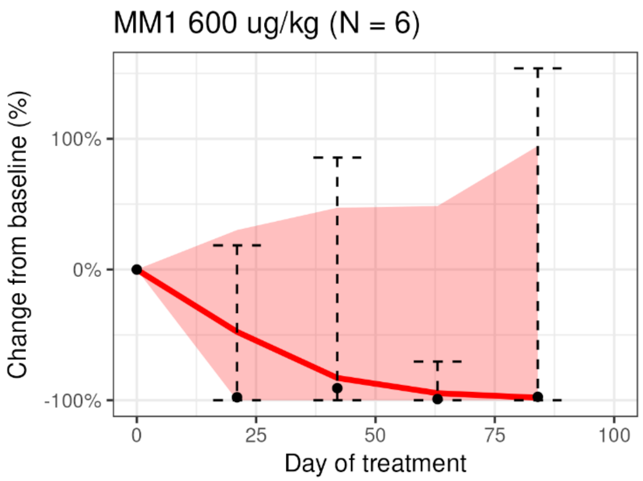

Supplementary Figure 9 MM1 spider plots for one dose escalation arm. Shown VPop is the same as that shown in Figure 2c. This study arm was substantially smaller regarding study size, with 6 participants compared to MM3-Cohort A large study (N = 120 participants). VPop shows that observed variability was captured at most observed points. Median change from baseline and 95% prediction intervals of a VPop are shown in the red solid line and shaded area, respectively, compared to the observed median and 95% coverage of observed values shown in black dots and error bars, respectively.

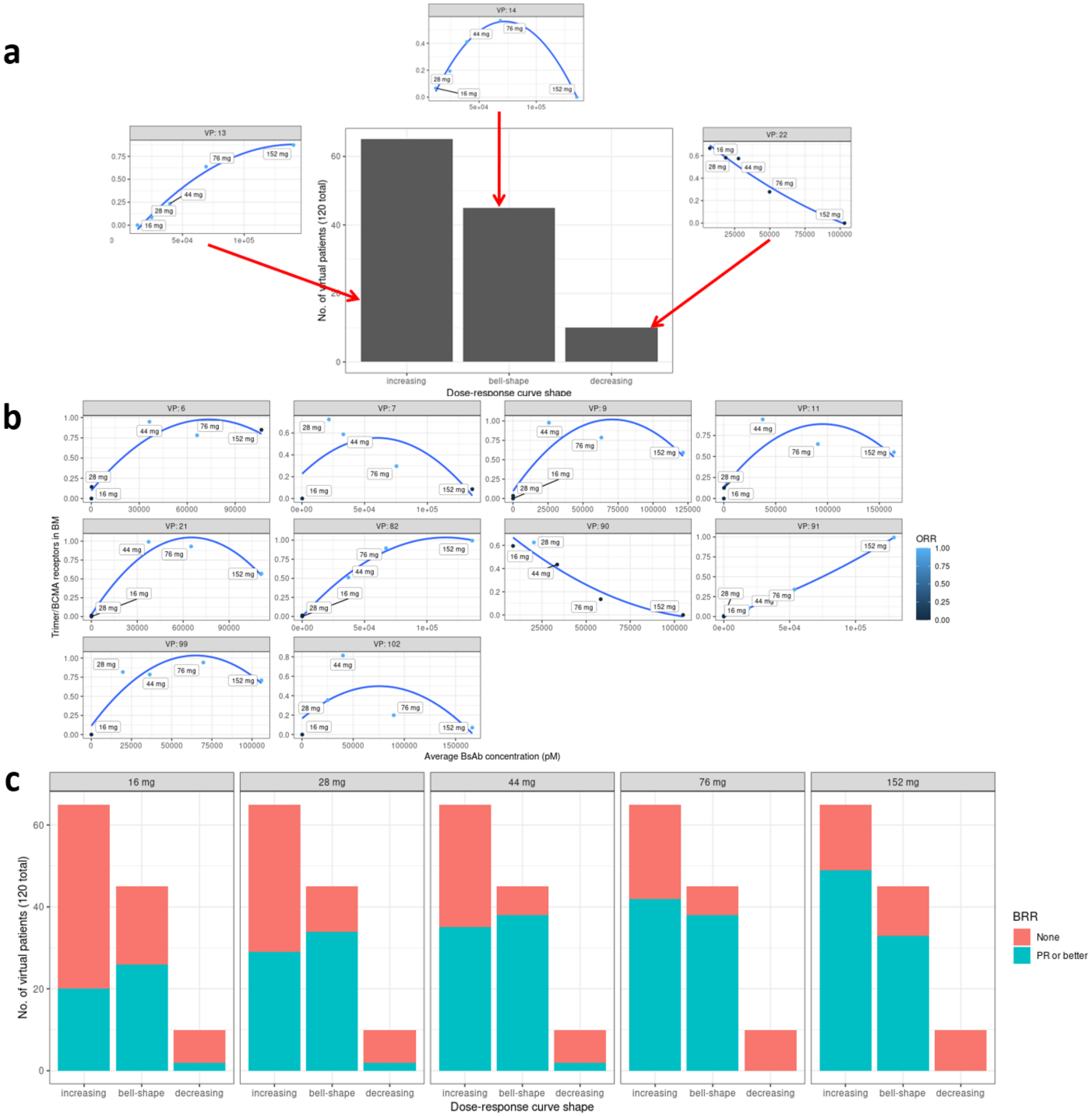

Supplementary Figure 10 Individual simulated dose-response curves are used to stratify patients into 3 *types*: increasing (patients who will have higher trimer:BCMA ratio as drug increases), decreasing (patients who will have higher trimer:BCMA ratio as drug decreases), or bell-shape. A) Majority of virtual patients fall into the first category, whereas less than 10% will have decreasing dose-response curve. B) Additional dose-response curves in virtual patients, each dose is colored by simulated biochemical response (blue = response, black = no response) for the virtual patient in panel. Virtual

patients 6 and 7 are classified as bell-shaped, and they interestingly will reach a biochemical response with doses 44 – 76 mg QW but did not reach a response at 16 mg QW or 152 mg QW. C) BRR across doses for each dose-response curve type shows that the increasing group has better BRR as dose increases, decreasing type has worse BRR, but bell-shaped curve type reaches maximum BRR at 76 mg QW.

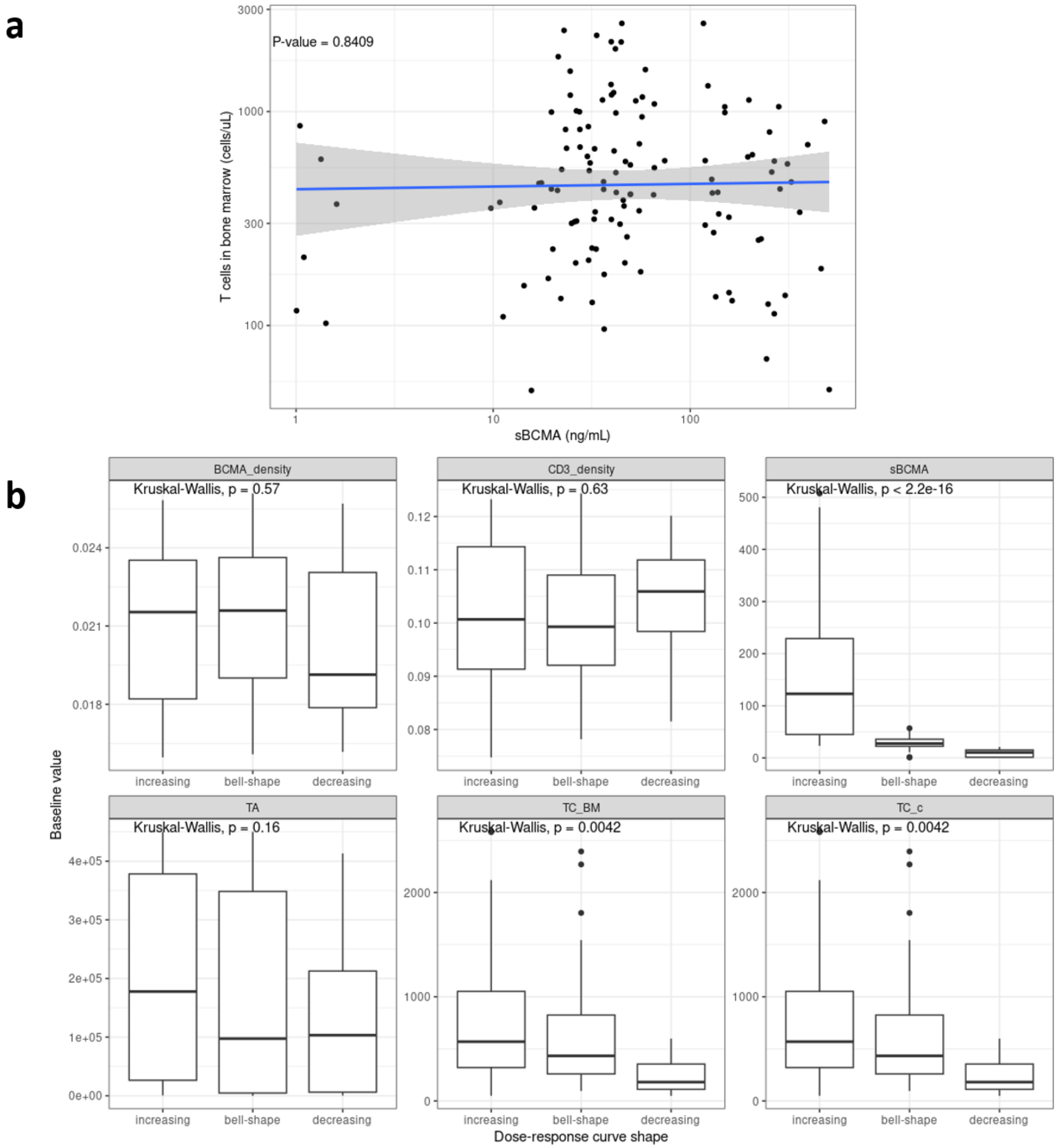

Supplementary Figure 11 Factors that were identified as the most statistically significant simultaneously for predicting dose-response curve type are A) sBCMA at baseline, B) T cell concentration at baseline, and C) α_{kill}, kill constant parameter that was perturbed across virtual patients. Further exploration of the relationship between sBCMA and T cells showed that, as expected, these variables were not correlated, suggesting that both these factors are important and independently

influence response. Higher levels of these factors seem to be associated with an increasing dose-response type, but it is important to note that too much sBCMA resulted in no response despite having an increasing dose-response curve type. Small levels of T cell concentration resulted in a decreasing dose-response curve type, suggesting low to no efficacy as shown in Supplementary Figure 10b. Thus, if a theoretical patient has low BL sBCMA, our model suggests that T cell concentrations, which are measurable in clinic, can also affect response levels, with lower levels lowering the chance of efficacy despite patient to be considered "low risk" due to sBCMA levels. Unfortunately, parameters such as α_{kill} are not measurable in clinic, suggesting additional unobserved tumor efficacy variables in clinic that one may not account for during dose selection.

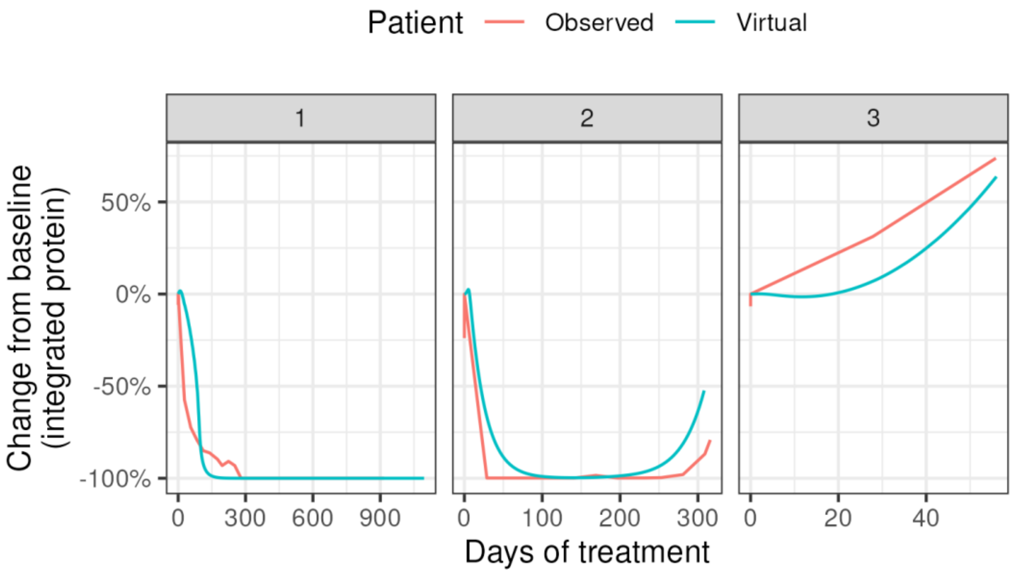

Supplementary Figure 12 Virtual patients (red) matched to observed patient's trajectories (blue). Three participants from the MM-3 study with different paraprotein trajectories were identified and matched to three virtual patients from one of the 10 VPops. Panel #1 corresponds to an observed patient and a matched virtual patient who showed early response and maintained it after more than 2.5 years of treatment. Panel #2 shows that both observed and virtual patient had progressive disease after day 300 due to paraprotein elevations. Lastly, panel #3 shows an observed and a matched virtual patient that did not show a response and were removed from the trial due to progression after day 50 with a similar relative increase in paraprotein levels.

Supplementary Table 1: Parameters Varied to generate PP

Parameter Name	Definition	Units	Default Value	VPop Range (LSA)	Reference
$CD3_{\text{density}}$	Density of CD3 receptors on T cells	Receptor/cell	6×10^4	±25%	[5]
k_{resist}	Rate of resistance maximal effect on trimer kill EC_{50} due to weakened T cell activation	1/hr	0.1	±2 fold	Calibrated
α_{resist}	Maximal effect of resistance on trimer kill EC_{50} due to weakened T cell activation	hr	0.5	±2 fold	Calibrated
$\mathcal{BCMA}_{\text{density}}$	Density of BCMA receptors on tumor cell	Receptors/cell	1.259×10^4	±25%	[3]
k_{exp}	Exponential-phase tumor growth rate	1/hr	3.32×10^{-4}	±10 fold	[5, 7]
k_{lin}	Linear-phase tumor growth rate	Cells/hr	1500	±10 fold	[7]
τ_{MM}	Transit time between tumor cell death compartments	hr	27	24–30	[8]
α_{kill}	Trimer tumor kill rate constant	1/hr	0.02	±10 fold	Calibrated
n_{kill}	Trimer tumor kill rate Hill coefficient	unitless	0.8	0.5–1.5	Calibrated

Supplementary Table 1: Parameters Varied to Generate PP. Each parameter was independently sampled from the biophysical ranges using uniform distributions.

Supplementary Table 2: Variables and Initial Conditions

Variable	Code Alias	Init. Value or [VPop Range]	Definition	Units
Ab_{sc}	Ab_sc	0	Free drug in sub-cutaneous compartment	pM
Ab_c	Ab_c	0	Free drug in central compartment	pM
Ab_{BM}	Ab_BM	0	Free drug in bone marrow compartment	pM
Tc_c	TC_c	[16 to 2,580]	T cells in central compartment	$cells/uL$
Tc_{BM}	TC_BM	[16 to 2,580]	T cells in bone marrow compartment	$cells/uL$
$sBCMA_c$	sBCMA	[185 to 153,520]	Soluble BCMA in central compartment	pM
$sBCMA_{BM}$	sBCMA_BM	[185 to 153,520]	Soluble BCMA in bone marrow compartment	pM
Ab_sBCMA_c	DsBCMA	0	Drug-sBCMA complex in central compartment	pM
Ab_CD3_c	DCD3_c	0	Drug-T cell dimer in central compartment	pM
Ab_sBCMA_{BM}	DsBCMA_BM	0	Drug-sBCMA complex in tumor compartment	pM
Ab_BCMA_{BM}	DBCMA	0	Drug-MM cell dimer in tumor compartment	pM
Ab_CD3_{BM}	DCD3_BM	0	Drug-T cell dimer in tumor compartment	pM
$Trimer$	Trimer_BM	0	Trimer between CD3, BCMA, and BsAB	pM
MM	TA	[0 to 450,000].	MM cells in BM	$cells/uL$
MM_i	TAi	0	MM cells through (i=2-4) transit compartments	$cells/uL$
M_P	MProtein	[0 to 70]	M-Protein in circulation	g/L
FLC	FLC	[0.92 to 174680]	FLC in circulation	mg/L
C_{BM}	cytokine_BM	0	Cytokines shed by T cells after trimer	pg/mL
C_i	Cytokine_i	0	Cytokines through transit compartments ($i = 1 - 5$)	pg/mL
C_c	Cytokine_c	2.857	Cytokines in central compartment	pg/mL

Supplementary Table 2: Model variables in QSP model. Initial conditions of drug concentration, dimers and trimer are set to zero, unless they are sampled from the reported range for PP generation. The distributions for the varied initial states were fitted to Pfizer baseline clinical data or obtained from literature, as described in QSP PMAR−1513. Initial values of the varied states (Tc_c, Tc_{BM}, $sBCMA_c$, $sBCMA_{BM}$, MM, M_p and FLC) were sampled from ranges shown in square brackets.

Supplementary Table 3: Full Table of Parameters

Section in QSP Model	Name [Code Alias]	Description	Units	Value	Source
**Sect. 1.1: BsAB Reactions Central**					
	k_{on1} [kon_BCMA]	Binding of BsAB to BCMA	$pM^{-1}hr^{-1}$	0.0054	Fixed internal nonclinical data
	k_{off1} [koff_BCMA]	Unbinding rate	hr^{-1}	0.162	Fixed internal nonclinical data
	k_{on2} [kon_CD3]	Binding of BsAB to CD3	$pM^{-1}hr^{-1}$	0.00198	Fixed internal nonclinical data
	k_{off2} [koff_CD3]	Unbinding rate	hr^{-1}	82.17	Fixed internal nonclinical data
	k_{deg_sBCMA} [kdeg_sBCMA]	Degradation of sBCMA	hr^{-1}	0.01925	Fixed internal nonclinical data
	ktr_{c_BM} [k_Ab_c_BM]	BsAB transport rate from central to BM	hr^{-1}	0.1	Estimated with clinical data
	ktr_{BM_c} [k_Ab_BM_c]	BsAB transport rate from BM to central	hr^{-1}	0.3	Estimated with clinical data
	$ktr_{sBCMA\_BM\_c}$ [k_sBCMA_BM_c]	Ab_sBCMA dimer transport rate from BM to central	hr^{-1}	0.1	Assumed equal to ktr_{c_BM}
	k_{el} [kel]	Elimination rate of BsAB and sBCMA dimer	hr^{-1}	CL/V_c	Preliminary PopPK
	k_a [ka]	Drug absorption rate	hr^{-1}	6.167E-3	Preliminary PopPK
	F [F_SC]	Drug bioavailability		0.511	Preliminary PopPK
	V_c [V1]	Volume in central	L	4.25	Preliminary PopPK
	V_p [V2]	Volume of peripheral	L	7.8	Preliminary PopPK
	Q [Q]	Inter-compartmental clearance	L/hr	0.008416	Preliminary PopPK
	Cl [CL]	Clearance	mL/hr	13.9583	Preliminary PopPK
**Sect. 1.2: T cell & Cytokines Central**					
	$k_{TC\_c\_BM}$ [k_TC_c_BM]	T cell transport from central to BM	hr^{-1}	0.2	Estimated with clinical data and literature [14]
	$k_{TC\_BM\_c}$ [k_TC_BM_c]	T cell transport from BM to central	hr^{-1}	0.3893	Estimated with clinical data and literature [14]
	k_{el_T} [kel_TC_c]	T cell elimination rate in central	hr^{-1}	0.1046	Fixed Literature [1]
	β_{prod} [basal_production]	Basal cytokine production	$pg/mL/hr$	1	Estimated literature [14]
	k_{deg_cyt} [cr_kdeg]	Degradation of cytokines	hr^{-1}	0.5	Estimated with clinical data and literature [14]
	ktr_{cyt} [k_tr_cyt]	Cytokine transit rates from BM to central	hr^{-1}	0.625	Estimated with clinical data and literature [14]
**Sect. 1.3: Paraproteins Central**					
	$k_{deg\_M_p}$ [MProtein_deg]	Degradation rate of M-protein	hr^{-1}	0.0021	Fixed Literature [12]
	k_{deg_FLC} [FLC_deg]	Degradation rate of FLC	hr^{-1}	0.1733	Fixed Literature [13]
**Sect. 2.2: Tumor Cells BM**					
	V_{BM}	Volume of bone marrow compartment	L	1.75	Fixed Literature [11, 10]

	k_{g0} [kg0]	Exponential tumor growth rate	hr^{-1}	3.32×10^{-4}	Calibrated with clinical data and literature [6, 4, 7]
	k_{g1} [kg1]	Linear tumor growth rate	$cells/hr$	1500	Calibrated with clinical data and literature [6, 7]
	ψ [growth_switch]	Switch between exponential and linear growth phases		20	Fixed Literature [9]
	α_{kill} [alpha_kill]	Kill constant	hr^{-1}	0.02	Calibrated with clinical data
	k_m [km_kill]	Half saturation for tumor killing	pM/cells/uL	4.25E-4	Estimated with clinical data and literature [14]
	n_{kill} [n_kill]	Cooperativity coefficient kill constant		0.8	Calibrated with clinical data
	τ_M [tau_TA]	Transit time to MM cell death	hr	24	Calibrated with clinical data and literature [8]
	MM_{max}	Maximum tumor burden	$cells/uL$	3×10^6	Estimated with clinical data
	α_{resis}[alpha_resis]	Maximum addition resistance		0.5	Calibrated with clinical data
	β_{resis} [beta_resis]	Rate of change of resistance	hr^{-1}	0.1	Calibrated with clinical data
	τ_{resis} [tau_resis]	Time to resistance	hr	600	Estimated with clinical data
	$BCMA_{\text{density}}$[BCMA_density]	Density of BCMA receptors per tumor cell	receptor/cell	12590	Calibrated with clinical data and literature [3]
**Sect. 2.3: T Cell & Cytokines BM**					
	$CD3_{\text{density}}$[CD3_density]	Density of CD3 receptors per T cell	receptor/cell	6×10^4	Calibrated with clinical data and literature [5]
	I_{max} [cr_Imax]	Maximum cytokine inhibition on cytokine release		1	Fixed Literature [2]
	n_{1C} [cr_n_IH]	Cooperativity coefficient for cytokine release inhibition		2.5	Fixed Literature [2]
	IC_{50}[cr_IC50]	Cytokine concentration resulting in 50% release inhibition	$pg/mL \times hr$	10000	[2]
	K [cr_K]	Priming factor		2.83	[2]
	N [n_dose_count]	Number of doses		1	[2]
	E_{max}[cr_Emax]	Maximum cytokine release rate	$pg/mL/hr$	80590	[2]
	EC_{50} [cr_EC50]	Exposure of active drug species to achieve 50% of E_max	pM	0.5	[2]
	n_{2C}[cr_n]	Hill coefficient for cytokine release		1	[2]
	I_{maxTC} [Imax_TC]	Maximum T cell migration inhibition		1	Estimated
	K_1 [ksat_TC]	Cytokine concentration at 50% of I_{maxTC}	pg/mL	150	Estimated
	K_2 [ksat_Trimer_TC]	Trimer concentration at 50 % of I_{maxTC}	pM	0.02	Estimated
	n_{cyt} [n_cytokine_TC]	Hill coefficient T cell transport inhibition from cytokines		2	Estimated
	n_{tri} [n_Trimer_TC]	Hill coefficient T cell transport inhibition from trimers		1.5	Estimated

Supplementary Table 3: Parameters in QSP model.

Supplementary Table 4: Study Data used for Model Calibration

**Study (N)**	**Elranatamab Dosing Regimen**	**Day(s) of sampling (SPEP, serum FLC)**	**Day(s) of sBCMA and PK Sampling**
C1071001 Part 1 (23)	IV: 0.1, 0.3, 1, 3, 10, 30, and 50 μg/kg QW	Screening: -14 to 1	
Cycle 1: 1	Cycle 1: 1		
C1071001 Part 1 (30)	SC: 80, 130, 215, 360, 600, and 1000 μg/kg QW	Screening: -14 to 1	
Cycle 1 onwards: 1	Cycle 1: 1, 8, 15		
Cycle 2: 1, 8, 15			
Cycle 3 onwards: 1			
C1071001 Part 1 C-D (13)	SC: combination	Screening: -14 to -1	
Cycle 1: 1	Cycle 1: 1		
C1071001 Part 1.1 QW (7)	SC: 600 μg/kg for the first week, then 1000 μg/kg QW	Screening: -14 to -1	
Cycle 0: 0			
Cycle 1 onwards: 1	Cycle 0: 0		
Cycle 1: 1, 8, 15, 22			
Cycle 2: 1, 8, 15, 22			
Cycle 3 onwards: 1			
C1071001 Part 1.1 Q2W (13)	SC: 600 μg/kg for the first week, then 1000 Q2W	Screening: -14 to -1	
Cycle 0: 0	Cycle 0: 0		
C1071001 Part 2A[a] (15)	SC: 44 mg for the first week, then 76 mg QW	Screening: -14 to -1	
Cycle 0: 0			
Cycle 1 onwards: 1	Cycle 0: 0		
Cycle 1: 1, 8, 15, 22			
Cycle 2: 1, 8, 15, 22			
Cycle 3 onwards: 1			
C1071003[a] Part A (120)	SC: 12/32/76 mg C1D1/C1D4/C1D8 then 76 mg QW[b]	Screening: -28 to -1	
Cycle 1 onwards: 1	Cycle 1: 1, 4, 8, 15, 22		
Cycle 2, 3, 4, 7, 10, etc.: 1			

Supplementary Table 4: Source: Protocol for Study C1071001, Protocol for Study C1071003.
[a] Premedication with acetaminophen, diphenhydramine, and dexamethasone.
[b] 76 mg Q2W after C6 with persistent response, 76 mg Q4W after C13.
Table abbreviations: N = number of participants; IV = intravenous; PK = pharmacokinetic; RRMM = relapsed/refractory multiple myeloma; PD = pharmacodynamic; QW = once a week; SC = subcutaneous.

Supplementary Note 1

1 QSP Model Description and Assumptions

1.1 Central compartment

In the central compartment, the key interactions captured by the model are binding of BsAB to shed sBCMA receptors in circulation, as well as to circulatory T cell CD3 receptors. In addition, circulatory paraproteins (M-protein, FLC) and generic cytokine reactions are also included in this compartment. Serum FLC and M-protein dynamics are given by a balance of synthesis rates, proportional to total tumor burden, and degradation rates.

1.1.1 BsAB binding kinetics in central compartment

The free drug, corresponding to unbound BsAB, denoted by $[Ab_c]$ can flow in from a subcutaneous compartment in the case of SC modality and also exchange with a peripheral compartment $[Ab_p]$. Free drug can bind either to unbound soluble BCMA receptors ($[sBCMA_c]$) or to free circulating CD3+T cell receptors ($[CD3_c]$).

We outline a few assumptions made to reduce the number of parameters:

- The free drug, corresponding to unbound BsAB, denoted by $[Ab_c]$ can flow in from a subcutaneous compartment in the case of SC modality and also exchange with a peripheral compartment $[Ab_p]$.
- Free drug can bind either to unbound soluble BCMA receptors ($[sBCMA_c]$) or to free circulating CD3+T cell receptors ($[CD3_c]$).
- The elimination rate for the unbound bispecific, $[Ab_c]$ and bispecific sBCMA dimers are assumed to be the same k_{el}.
- Similar to work of [1], a trimer between sBCMA and T cells in circulation is omitted from the model equations since this species is not pharmacologically active.
- Synthesis of sBCMA in the central compartment is assumed to be proportional to the amount of sBCMA that enters the central compartment from the bone marrow and the system was in steady state at baseline ($t = 0$).

Thus the equations in their final form read:

$$
\begin{aligned}
\frac{d[Ab_c]}{dt} &= \frac{Input_{iv}}{V_c} + \frac{Fk_a[Ab_{sc}]}{V_c} - k_{12}[Ab_c] + k_{21}\frac{V_p}{V_c}[Ab_p] \qquad (1)\\
&- k_{on1}[Ab_c][sBCMA_c] + k_{\text{off}1}[Ab\_sBCMA_c] - k_{on2}[Ab_c][CD3_c] + k_{\text{off}2}[Ab\_CD3_c]\\
&- ktr_{c\_BM}[Ab_c] + ktr_{BM\_c}\frac{V_{BM}}{V_c}[Ab_{BM}] - k_{el}[Ab_c]\\
\frac{d[sBCMA_c]}{dt} &= k_{deg\_sBCMA}\left(\frac{[sBCMA_c^0]}{[sBCMA_{BM}^0]}[sBCMA_{BM}] - [sBCMA_c]\right) \qquad (2)\\
&- k_{on1}[Ab_c][sBCMA_c] + k_{\text{off}1}[Ab\_sBCMA_c] \qquad (3)\\
\frac{d[Ab\_sBCMA_c]}{dt} &= k_{on1}[Ab_c][sBCMA_c] - k_{\text{off}1}[Ab\_sBCMA_c] \qquad (4)\\
&+ ktr_{sBCMA\_BM\_c}\frac{V_{BM}}{V_c}[Ab\_sBCMA_{BM}] - k_{el}[Ab\_sBCMA_c]\\
\frac{d[Ab\_CD3_c]}{dt} &= k_{on2}[Ab_c][CD3_c] - k_{\text{off}2}[Ab\_CD3_c] \qquad (5)
\end{aligned}
$$

In the next section we outline how the CD3 receptor counts are obtained for BsAB-T cell dimer formation.

1.1.2 CD3+T cell and cytokine dynamics in the central compartment

We make the following assumptions:

- CD3+T cells in the central compartment, denoted by $[Tc_c]$ can either flow out of the central compartment into tissues (peripheral) and tumor site (BM), be eliminated, or generate at a balanced rate proportional to initial circulating T cell counts (Tc_c^0).
- Pro-inflammatory cytokines ($[C_c]$), e.g. IL-6, flow into the central compartment from the BM, when trimers are activated.
- The individual T cell level receptor production is constant and set at literature obtained CD3 receptor values, similar to [1].

Accordingly, we write the following ODE for T cells and cytokines in circulation:

$$
\begin{aligned}
\frac{d[Tc_c]}{dt} &= k_{el\_T}[Tc_c^0] - (k_{el\_T} + k_{TC\_c\_BM})[Tc_c] + k_{TC\_BM\_c}[T_{BM}]\frac{V_{BM}}{V_c}(1 - T_{inh}) \qquad (6)\\
\frac{d[C_c]}{dt} &= \beta_{prod} + k_{tr\_cyt}[C_5]\frac{V_{BM}}{V_c} - k_{deg\_cyt}[C_c]. \qquad (7)
\end{aligned}
$$

We define T_{inh} term which controls T cell outflow from site of action in section 1.2.3.

To obtain the number CD3 receptors in the central compartment:

$$
\begin{aligned}
CD3_{tot\_c} &= [Tc_c] \times CD3_{density}\frac{L\_to\_uL}{av\_num}1e12 \qquad (8)\\
CD3_c &= CD3_{tot\_c} - Ab\_CD3_c \qquad (9)
\end{aligned}
$$

The initial conditions for these additional equations are:

$$[Tc_c(0)] = Tc_c^0, \quad [CD3_c(0)] = [Tc_c^0] \times CD3_{density} \frac{L\_to\_uL}{av\_num} 1e12, \tag{10}$$

which supports the dimer initial condition mentioned in the prior section, namely:

$$[Ab\_CD3_c(0)] = 0.$$

1.1.3 Paraproteins in the central compartment

Similar to the assumptions made for sBCMA, we make these assumptiosn for the paraprotein dynamics:

- Basal M-spike and FLC synthesis in circulation is set to zero.
- Paraprotein production is driven by tumor burden since the synthesis rate is proportional to total tumor burden in BM, $[MM_{tot}]$.
- Amount of paraprotein entering the central compartment from the bone marrow was in steady state at $t = 0$.

Thus, the model equations for paraproteins in the central compartment are:

$$\frac{d[M_P]}{dt} = k_{deg\_Mp} \frac{[M_P^0]}{[MM_{tot}^0]} [MM_{tot}] - k_{deg\_Mp}[M_P] \tag{11}$$

$$\frac{d[FLC]}{dt} = k_{deg\_FLC} \frac{[FLC^0]}{[MM_{tot}^0]} [MM_{tot}] - k_{deg\_FLC}[FLC]. \tag{12}$$

where $MM_{tot} = [MM] + [MM_2] + [MM_3] + [MM_4]$ is the total tumor burden. Initial conditions are $M_P(0) = M_P^0, FLC(0) = FLC^0$.

1.2 Bone marrow (BM) compartment

In the bone marrow (BM) compartment, the key interactions captured by the model are binding of BsAb to shed sBCMA receptors in BM, membrane-bound BCMA receptors, and T cell CD3 receptors. A functional trimer is formed when BsAb is simultaneously bound to the BCMA and CD3 receptors, and this complex drives tumor-killing in the BM and cytokine release.

1.2.1 Drug kinetics in Bone Marrow

We start with the following assumptions in the BM:

- The rate of BCMA shedding is assumed to be proportional to tumor burden.
- As assumed with other model states, the system is at steady state at $t = 0$.
- Central and BM binding rates are assumed to the the same
- Complex formation rates for sBCMA and BCMA are assumed to be the same (i.e., BsAb binds to sBCMA and BCMA at same rate).

The dynamics of free drug (BsAB) and sBCMA in the BM compartment are given by

$$\frac{d[Ab_{BM}]}{dt} = k_{\text{off1}}[Ab\_sBCMA_{BM}] - k_{on1}[Ab_{BM}][sBCMA_{BM}] \tag{13}$$
$$+ k_{\text{off2}}[Ab\_CD3_{BM}] - k_{on2}[Ab_{BM}][CD3\_BM] + k_{\text{off1}}[Ab\_BCMA_{BM}]$$
$$- k_{on1}[Ab_{BM}][BCMA_{BM}] - ktr_{BM\_c}[Ab_{BM}] + ktr_{c\_BM}\frac{V_c}{V_{BM}}[Ab_c]$$

$$\frac{d[sBCMA_{BM}]}{dt} = k_{deg\_sBCMA}\frac{V_c}{V_{BM}}\frac{[sBCMA_c^0]}{[MM_{tot}^0]}[MM_{tot}] - ktr_{sBCMA}[sBCMA_{BM}] \tag{14}$$
$$- k_{on1}[Ab_{BM}][sBCMA_{BM}] + k_{\text{off1}}[Ab_sBCMA_{BM}]$$

The rest of the dimerization and trimer formation equations are:

$$\frac{d[Ab\_BCMA_{BM}]}{dt} = k_{on1}[Ab_{BM}][BCMA_{BM}] - k_{\text{off1}}[Ab\_BCMA_{BM}] \tag{15}$$
$$- k_{on2}[Ab\_BCMA_{BM}][CD3_{BM}] + k_{\text{off2}}[Trimer]$$

$$\frac{d[Ab\_sBCMA_{BM}]}{dt} = k_{on1}[Ab_{BM}][sBCMA_{BM}] - k_{\text{off1}}[Ab\_sBCMA_{BM}] \tag{16}$$
$$- ktr_{sBCMA\_BM\_c}\frac{V_{BM}}{V_c}[Ab\_sBCMA_{BM}] \tag{17}$$

$$\frac{d[Ab\_CD3_{BM}]}{dt} = k_{on2}[Ab_{BM}][CD3_{BM}] - k_{\text{off2}}[Ab\_CD3_{BM}] \tag{18}$$
$$- k_{on1}[Ab\_CD3_{BM}][BCMA_{BM}] + k_{\text{off1}}[Trimer]$$

$$\frac{d[Trimer]}{dt} = k_{on1}[Ab\_CD3_{BM}][BCMA_{BM}] - k_{\text{off1}}[Trimer] \tag{19}$$
$$+ k_{on2}[Ab\_BCMA_{BM}][CD3_{BM}] - k_{\text{off2}}[Trimer]$$

The total receptor counts in the bone marrow are given by

$$CD3_{totBM} = Tc_{BM} \times CD3_{density}\frac{L\_to\_uL}{av\_num}1e12 \tag{20}$$
$$BCMA_{totBM} = MM_{tot} \times BMCA_{density}\frac{L\_to\_uL}{av\_num}1e12 \tag{21}$$

and the free receptors in the bone marrow are calculated:

$$BCMA_{BM} = BCMA_{totBM} - Ab\_BCMA_{BM} - Trimer \tag{22}$$
$$CD3_{BM} = CD3_{totBM} - Ab\_CD3_{BM} - Trimer. \tag{23}$$

1.2.2 Tumor cell dynamics in BM

The equations for tumor plasma cell dynamics follow the standard Simeoni [9] tumor growth dynamics with a kill term driven by trimer concentration as follows:

$$\frac{d[MM]}{dt} = k_{g_0} \frac{1 - \frac{[MM_{tot}]}{MM_{max}}}{\left(1 + \left(\frac{k_{g_0}}{k_{g_1}} MM_{tot}\right)^{\psi}\right)^{1/\psi}} [MM] - K_{\mathrm{Mkill}}[MM] \tag{24}$$

$$\frac{d[MM_2]}{dt} = K_{\mathrm{Mkill}}[MM] - \frac{4}{\tau_M}[MM_2] \tag{25}$$

$$\frac{d[MM_3]}{dt} = \frac{4}{\tau_M}[MM_2] - \frac{4}{\tau_M}[MM_3] \tag{26}$$

$$\frac{d[MM_4]}{dt} = \frac{4}{\tau_M}[MM_3] - \frac{4}{\tau_M}[MM_4] \tag{27}$$

where τ_M is a total delay transit compartment rate. The rate of tumor killing and the overall efficacy is dependent on the trimer to tumor ratio as follows:

$$K_{\mathrm{Mkill}} = \alpha_{kill} \frac{(\frac{[Trimer]}{\varepsilon + [MM]})^{n_{kill}}}{k_{mkill}^{n_{kill}} + (\frac{[Trimer]}{\varepsilon + [MM]})^{n_{kill}}}.$$

The term ε is a small value introduced for numerical stability. With this formulation, tumor killing would be achieved if trimer levels are high while tumor levels are either low or high, or when trimer and tumor levels are both low.

Finally, the tumor killing half-max effect term, k_{mkill} was designed to be time-dependent in order to capture the patients who showed initial FLC/M-protein lowering but then ultimately relapsed with an increase in paraproteins. This weakening of trimer kill efficacy over time can correspond to eventual attenuation of T cell activity due to variety of known mechanisms, such as PD-1 expression. We implement this effect using the following equation:

$$k_{mkill} = k_m \left(1 + \alpha_{resis}(1 - e^{-\beta_{resis}(t - \tau_{resis})}\right)$$

where α_{resis} is the maximum addition resistance that increases the trimer/drug needed to induce efficacy, τ_{resis} is the time it takes for the resistance to occur, and β_{resis} is the rate at which the resistance reaches maximum effect.

1.2.3 CD3+T cell and cytokine dynamics in BM

For BM T cell and cytokine dynamics, we partially adapt the model of [2] with key focus on production of a pro-inflammatory cytokine (e.g., IL-6) in response to T cell activation due to binding in a trimer complex with MM cells in the BM. Additional assumptions or deviations from Chen et al. include:

- Significant T cell activation in the BM results in inhibition of T cell back-flow from the BM back into circulation.
- Cumulative cytokine exposure is reduced with each new dosing interval, instead of being set to 0.

As in the central compartment, we have an equivalent BM equation for T cells:

$$\frac{d[Tc_{BM}]}{dt} = k_{TC\_c\_BM}\frac{V_c}{V_{BM}}[Tcells_c] - k_{TC\_BM\_c}[Tc_{BM}]\,(1 - T_{inh}) \tag{28}$$

with a T cell transport inhibition term,

$$T_{inh} = I_{max\,TC}\left(\frac{\bar{C}_{BM}^{n_{cyt}}}{K_1^{n_{cyt}} + \bar{C}_{BM}^{n_{cyt}}}\right)\left(\frac{Trimer^{n_{tri}}}{K_2^{n_{tri}} + Trimer^{n_{tri}}}\right). \tag{29}$$

The quantity $\bar{C}_{BM} = (C_{BM} + C_1 + C_2 + C_3 + C_4 + C_5)/6$ gives the average cytokine levels in the BM. Thus, T cells transport out of the BM decreases as BM trimer and cytokine levels increase.

The cytokine equations read as follows:

$$\frac{d[C_{BM}]}{dt} = R_{syn}(1 - IH) - (k_{tr\_cyt} + k_{deg\_cyt})[C_{BM}] \tag{30}$$

$$\frac{d[C1]}{dt} = k_{tr\_cyt}[C_{BM}] - (k_{tr\_cyt} + k_{deg\_cyt})[C1] \tag{31}$$

$$\frac{d[C2]}{dt} = k_{tr\_cyt}[C1] - (k_{tr\_cyt} + k_{deg\_cyt})[C2] \tag{32}$$

$$\frac{d[C3]}{dt} = k_{tr\_cyt}[C2] - (k_{tr\_cyt} + k_{deg\_cyt})[C3] \tag{33}$$

$$\frac{d[C4]}{dt} = k_{tr\_cyt}[C3] - (k_{tr\_cyt} + k_{deg\_cyt})[C4] \tag{34}$$

$$\frac{d[C5]}{dt} = k_{tr\_cyt}[C4] - (k_{tr\_cyt} + k_{deg\_cyt})[C5] \tag{35}$$

$$\frac{d[C_{auc}]}{dt} = [C_{BM}] - \beta_{prod} \tag{36}$$

The terms $[C_{BM}]$ to $[C_5]$ represent cytokine concentrations in various compartments (i.e. $[C_{BM}]$: release compartment, $[C_1]$ to $[C_5]$: five transit compartments, $[C_c]$: circulation compartment described in the central compartment section).

Similar to [2] a negative feedback loop, IH was incorporated by assuming inhibition on cytokine release is a function of cumulative cytokine exposure during individual dosing intervals, C_{auc}, whereas cytokine release rate, R_{syn} is trimer dependent. The cumulative cytokine exposure in this model is reduced (by division) at each dosing interval using a Hill equation dependent on a dose counter:

$$C_{auc,N}(0) = \frac{C_{auc,N-1}(\tau)}{5\left(1 - \frac{(N+1)^2}{1.3^2+(N+1)^2}\right)} \tag{37}$$

where N is the number of doses and τ is the length of the dosing interval.

Thus, the equations for cytokine release inhibition and cytokine release are given by

$$IH = I_{max}\frac{C_{auc}^{n_{1C}}}{IC_{50}^{n_{1C}} + C_{auc}^{n_{1C}}} \qquad R_{syn} = E_{max}\frac{Trimer^{n_{2C}}}{EC_{50}^{n_{2C}} + Trimer^{n_{2C}}}. \tag{38}$$

1.3 Description of objective function for virtual population calibration

From the plausible population, we select 120 virtual patients by using an objective function that uses the 3 metrics described below. All endpoints described are used simultaneously for model calibration and applied via a genetic algorithm (see Figure S3a for genetic algorithm flowchart).

1.3.1 Best biochemical objective response

Best biochemical objective respose (BOR) is defined as the greatest decrease of percent change from baseline of integrated paraproteins (serum M-protein or FLC). We use BOR from the MM3 Cohort A trial ($n = 120$ patients) to calibrate the BOR of the virtual populations. Let $b_{(i)}$ be the ranked BOR of patients $i = 1, \ldots, 120$, we separatate the ranked BOR into 10 distinct groups and estimate the median BOR for each group, b_g for $g = 1, \ldots, 10$, as shown in Figure S6. We repeat the process with a virtual population of 120 virtual patients, $B_{(j)}$ where $j = 1, \ldots, 120$, and the median BORs for each group is B_g for $g = 1, \ldots, 10$. We minimize the function:

$$\sum_{g=1}^{10}(b_g - B_g)^2$$

and select the proposed virtual population (VP) such that the function above yields a value below an acceptable threshold.

1.3.2 Paraprotein dynamics

We define the paraprotein dynamics for patients i as the percent change from baseline in the integrated paraprotein (see Figure S4 for guideline of biomarker selection) in the form:

$$D_{i,t} = \frac{x_{i,t} - x_{i,0}}{x_{i,0}}$$

where $x_{i,t}$ is the value of the paraprotein at scheduled assessment t, and x_0 is the baseline value. We use the paraprotein dynamics from all monotherapy arms with QW dosing in both studies (MM1 and MM3 Cohort A) which results in 7 different arms. For each arm, $a = 1, \ldots, 7$, we obtain the median percent change at each tumor assessment point, t (every 3 weeks) and minimize the least squares, weighing by number of patients of each arm, n_a. Thus, our objective function is:

$$\sum_{a=1}^{7} n_a \sum_{t_a} (d_{median,t_a} - D_{median,t_a})^2$$

where d_{median,t_a} is the median percent change for a proposed virtual population (simulated under similar conditions as the arm it is being compared to) at tumor assessment time t_a in arm a.

1.3.3 Biochemical response rates

Biochemical response rates (BRR) are defined as the percent of patients in each arm that had a decrease of 50% of greater from baseline and response is observed for at least two consecutive tumor assessments. This metric is similar to the objective response rates, but relies on only

serum paraprotein responses. We used the same arms used for the paraprotein dynamics response for calibration but stratified patients by sBCMA levels at baseline (high vs low). Thus, we have up to 14 possible arms to calibrate BRR. Let $R_{a,h}$ be the BRR for patients in arm $a = 1, \ldots, 7$ and sBLCA group $h = 0(low\, sBCMA), 1(high\, sBCMA)$. We propose a virtual population, stratify by high/low levels of sBCMA, simulate all 7 arm schedules, and calculate BRR, $r_{a,h}$. We add the following term to the objective function:

$$\sum_{a=1}^{7}\sum_{h=0}^{1} n_{a,h}(r_{a,h} - R_{a,h})^2.$$

1.4 Miscellaneous

1.4.1 Calculation of ***standardized proportion of trimers*** from Figure 4

We take the simulated dynamics of drug, trimer and dimer (drug-BCMA) concentration of all virtual patients accross the five simulated schedules (16 mg, 28 mg, 44 mg, 76 mg, and 152 mg QW). For each dose and each patient, we calculate the mean drug concentration. Let $\bar{T}_i$ and $\bar{M}_i$ be the average trimer and drug-BCMA dimer concentration for patient i, respectively. For each dose, we calculate the ratio of trimers to BCMA bound complexes, $R_{trimer,i,dose}$:

$$R_{trimer,i,dose} = \frac{\bar{T}_{i,dose}}{\bar{T}_{i,dose} + \bar{M}_{i,dose}}$$

We standardize the ratio accross the 5 simulated for each patient:

$$R^*_{trimer,i,dose} = \frac{R_{trimer,i,dose} - \min_{dose=1,\ldots,5}(R_{trimer,i,dose})}{\max_{dose=1,\ldots,5}(R_{trimer,i,dose})} \tag{39}$$

Figure 4 shows the mean drug concentration plotted against $R^*_{trimer,i,dose}$ for 4 virtual patients.

Supplementary References

[1] Betts, A., Haddish-Berhane, N., Shah, D.K. et al. A Translational Quantitative Systems Pharmacology Model for CD3 Bispecific Molecules: Application to Quantify T Cell-Mediated Tumor Cell Killing by P-Cadherin LP DART. AAPS J 21, 66 (2019).

[2] Chen, X., Kamperschroer, C., Wong, G. and Xuan, D. (2019), A Modeling Framework to Characterize Cytokine Release upon T-Cell–Engaging Bispecific Antibody Treatment: Methodology and Opportunities. Clin Transl Sci, 12: 600-608 (2019).

[3] Friedman, K. M., Garrett, T. E., Evans, J. W., Horton, H. M., Latimer, H. J., Seidel, S. L., Horvath, C. J., & Morgan, R. A. (2018). Effective Targeting of Multiple B-Cell Maturation Antigen-Expressing Hematological Malignances by Anti-B-Cell Maturation Antigen Chimeric Antigen Receptor T Cells. Human gene therapy, 29(5), 585–601. https://doi.org/10.1089/hum.2018.001

[4] Hokanson, J A et al. Tumor growth patterns in multiple myeloma. Cancer vol. 39,3 (1977): 1077-84.

[5] Nicolas, L et al. Human gammadelta T cells express a higher TCR/CD3 complex density than alphabeta T cells. Clinical immunology (Orlando, Fla.) vol. 98,3 (2001): 358-63. doi:10.1006/clim.2000.4978

[6] Oivanen T. M. Plateau phase in multiple myeloma: an analysis of long-term follow-up of 432 patients. Finnish Leukaemia Group. Br J Haematol, 1996. 92(4): p. 834-9.

[7] Oivanen, T.M. Prognostic value of serum M-protein doubling time at escape from plateau of multiple myeloma. The Finnish Leukaemia Group. Eur J Haematol, 1996. 57(3): p. 247-53.

[8] Ramakrishnan, V. and D.E. Mager. Pharmacodynamic Models of Differential Bortezomib Signaling Across Several Cell Lines of Multiple Myeloma. CPT: Pharmacometrics & Systems Pharmacology, 2019. 8(3): p. 146-157.

[9] Simeoni M. et al. Predictive Pharmacokinetic-Pharmacodynamic Modeling of Tumor Growth Kinetics in Xenograft Models after Administration of Anticancer Agents. Cancer Res, 2004. 64 (3) p. 1094–1101

[10] Nombela-Arrieta C., Manz G. M, Quantification and three-dimensional microanatomical organization of the bone marrow. Blood Adv 2017; 1 (6): 407–416.

[11] Hassan HT, El-Sheemy M. Adult bone-marrow stem cells and their potential in medicine. J R Soc Med. 2004 Oct;97(10):465-71.

[12] Mills, JR et al. High sensitivity blood-based M-protein detection in sCR patients with multiple myeloma. Blood Cancer J. 2017 Aug; 7(8):e590. doi: 10.1038/bcj.2017.75. PMID: 28841203; PMCID: PMC5596386

[13] Tosi P, et al. Serum free light-chain assay for the detection and monitoring of multiple myeloma and related conditions. Ther Adv Hematol. 2013 Feb;4(1):37-41. doi: 10.1177/2040620712466863. PMID: 23610612; PMCID: PMC3629763.

[14] Topp M., Duell J. et al, Anti–B-Cell Maturation Antigen BiTE Molecule AMG 420 Induces Responses in Multiple Myeloma. Journal of Clinical Oncology 2020 38:8, 775-783. PMID: 31895611.

[15] Schropp, J., Khot, A., Shah, D. K., Koch, G. Target-Mediated Drug Disposition Model for Bispecific Antibodies: Properties, Approximation, and Optimal Dosing Strategy. CPT: Pharmacometrics & Systems Pharmacology, Vol. 8 No. 3, (2019).

[16] Mager, D. Krzyzanski, W. Quasi-Equilibrium Pharmacokinetic Model for Drugs Exhibiting Target-Mediated Drug Disposition. Pharmaceutical Research, Vol. 22, No. 10, (2005)
